# Knowledge management for systems biology a general and visually driven framework applied to translational medicine

**DOI:** 10.1186/1752-0509-5-38

**Published:** 2011-03-05

**Authors:** Dieter Maier, Wenzel Kalus, Martin Wolff, Susana G Kalko, Josep Roca, Igor Marin de Mas, Nil Turan, Marta Cascante, Francesco Falciani, Miguel Hernandez, Jordi Villà-Freixa, Sascha Losko

**Affiliations:** 1 Biomax Informatics AG, Planegg, Germany; 2 Hospital Clinic-IDIBAPS-CIBERES, Universitat de Barcelona, Barcelona, Spain; 3 School of Biosciences and Institute of Biomedical Research (IBR), University of Birmingham, Birmingham, UK; 4 Departament de Bioquimica i Biologia Molecular, Institut de Biomedicina at Universitat de Barcelona IBUB and IDIBAPS-Hospital Clinic, Barcelona, Spain; 5 Computational Biochemistry and Biophysics lab, Research Unit on Biomedical Informatics (GRIB) of IMIM/UPF, Parc de Recerca Biomdica de Barcelona (PRBB); Barcelona, Spain

## Abstract

**Background:**

To enhance our understanding of complex biological systems like diseases we need to put all of the available data into context and use this to detect relations, pattern and rules which allow predictive hypotheses to be defined. Life science has become a data rich science with information about the behaviour of millions of entities like genes, chemical compounds, diseases, cell types and organs, which are organised in many different databases and/or spread throughout the literature. Existing knowledge such as genotype - phenotype relations or signal transduction pathways must be semantically integrated and dynamically organised into structured networks that are connected with clinical and experimental data. Different approaches to this challenge exist but so far none has proven entirely satisfactory.

**Results:**

To address this challenge we previously developed a generic knowledge management framework, BioXM™, which allows the dynamic, graphic generation of domain specific knowledge representation models based on specific objects and their relations supporting annotations and ontologies. Here we demonstrate the utility of BioXM for knowledge management in systems biology as part of the EU FP6 BioBridge project on translational approaches to chronic diseases. From clinical and experimental data, text-mining results and public databases we generate a chronic obstructive pulmonary disease (COPD) knowledge base and demonstrate its use by mining specific molecular networks together with integrated clinical and experimental data.

**Conclusions:**

We generate the first semantically integrated COPD specific public knowledge base and find that for the integration of clinical and experimental data with pre-existing knowledge the configuration based set-up enabled by BioXM reduced implementation time and effort for the knowledge base compared to similar systems implemented as classical software development projects. The knowledgebase enables the retrieval of sub-networks including protein-protein interaction, pathway, gene - disease and gene - compound data which are used for subsequent data analysis, modelling and simulation. Pre-structured queries and reports enhance usability; establishing their use in everyday clinical settings requires further simplification with a browser based interface which is currently under development.

## Background

In biological or clinical research the creation of knowledge, here defined as "*the realisation and understanding of patterns and their implications existing in information*" relies on data mining. This in turn requires the collection and integration of a diverse set of up-to-date data and the associated context i.e. *information*. These sets include unstructured information from the literature, specifically extracted information from the multitude of available databases, experimental data from "-omics" platforms as well as phenotype information and clinical data. Although a large amount of information is stored in numerous different databases (the 2010 NAR database issue listing more than 1200 [1]) even more is still embedded in unstructured free text. Over the last 15 years a large number of methods and software tools have been developed to integrate aspects of biological knowledge such as signalling pathways or functional annotation with experimental data. However, it has proven extremely difficult to couple true semantic integration (i.e. the mapping of equivalent meaning and objects) across all information types relevant in a life science project with a flexible and extendible data model, robustness against structural changes in services and data, transparent usage, and low set-up and maintenance requirements (see [2] for an excellent recent review). In principle this difficulty arises from the high complexity of life science data, which is partly an artefact of the fragmented landscape of data sources but also stems from reasons integral to the life sciences. The ever extending "parts-list of life" itself already offers an astounding number of object classes, from the molecular to the organism, even if common naming/identifier and definitions could be agreed upon. In addition experimental data can only be interpreted in the context of the exact identity of the experimental sample, the samples environment, the samples processing and the processing and quality of the generated data. Even more than the occasional extension of the "parts-list" from our growing knowledge, technical development continually generates new data types, processing methods and experimental conditions. While life science projects in general will (hopefully) share some concepts, almost each one will require some individual adjustment to integrate and view the relevant information. Therefore an optimal data integration approach will ensure that the data model can be based on existing concepts (ideally ontological i.e. controlled, structured vocabulary) yet remains flexible and extendible by the advanced user. In this respect today's most successful (i.e. widely used) data integration approaches such as SRS [3] or Entrez [4] show only weak, cross-reference based data integration without semantic mapping to a common concept (categorised as link/index integration by Köhler [5] and Stein [6]). They depend on pairwise mappings between individual database entries provided by the data source e.g. from a protein sequence entry to the corresponding transcript, the mappings lack semantic meaning i.e. the notion that a protein is expressed from a gene can not be stated or queried. Additional processing and data mapping is required to answer even simple questions such as "which molecular mechanisms are known to be involved in the pathology of chronic obstructive pulmonary disease ?". Currently custom-developed data warehouses such as Atlas [7], BIOZON [8] or BioGateway [9], are the most common technical concept to achieve full semantic integration (in public and industry projects). While these are ideally suited to answer complex queries their inflexible and pre-determined data model and the necessary, often difficult, data synchronisation result in high set-up and maintenance costs. Further, adaptation of such data warehouses structure to an ever changing environment or requirements are difficult at best [6]. Fortunately, as more data sources start to adopt semantic web representations such as OWL [10] and RDF [11] maintenance for semantic mappings becomes less of an issue as concomitantly to adopting a common language to transport semantics many data sources also standardise the semantics they provide such as using common entity references and ontologies.

An optimisation, at least regarding data synchronisation, has been to present a semantically fully integrated view of the data while the underlying data is assembled on-the-fly from distributed sources using a coherent data model and semantic mappings [12,13] (categorised as federation/view integration by Köhler and Stein [5,6]). Details of this approach vary widely. The ad-hoc data assembly process can be provided by home made scripts or, more recently, using workflow engines such as Taverna [14]. The data model can be programmed with a specific language as in Kleisli [15] or may make use of standard ontologies as with TAMBIS [16]. Semantic mapping to the common concept can be produced by a view providing environment, such as BioMediator [17] and the Bio2RDF project [18], or can come from individual integrated data sources. In the latter case the data sources either provide such mappings voluntarily, working for the common good of the "semantic web" [19] or are forced to do so by a closed application environment such as caBIG [20], Gen2Phen/PaGE-OM [12,21] or GMOD [22]. While conceptually elegant, these approaches have some disadvantages: the start-up costs are quite high (e.g. [13,23]), the performance is determined by the slowest, least stable of the integrated resources, complex queries result in large joins which are hard to optimise, and data models are often hard to extend. Ad-hoc desktop data integration and visualisation tools such as Cytoscape [24], Osprey [25] or ONDEX [26] on the other hand combine excellent flexibility with good performance due to local data storage, however they do not allow large scale knowledge bases to be collaboratively generated, managed and shared.

Another issue, which is only partially addressed by current data integration solutions, is the need to organise not only public information but project-specific knowledge and data, keep it private or partially private for some time, store and connect experimental results and corresponding metainformation about materials and methods and, if eventually verified, merge it into the pool of common knowledge. This may for example take the form of an existing signal transduction pathway which is privately extended with new members or connections. The extension is then published and discussed within a specific project until it is accepted as common knowledge. While data resources such as GEO provide the option to keep submitted data private for some time, they generally do not allow existing knowledge to be extended as described above or allow existing data to be annotated with private or public comments.

Our challenge was to develop a knowledge management environment that achieves several goals: focus on the management of project-specific knowledge; ease data model generation and extension; provide completely flexible data integration and reporting methods combined with intuitive visual navigation and query generation; and address the issues of set-up and maintenance cost.

To do so we chose to apply different aspects of the approaches described above. In the next sections we describe the creation of a knowledge base for chronic diseases based on the BioXM software platform that efficiently models complex research environments with a flexible management, query and reporting interface which automatically adapts to the conceptualisation of the modelled information.

## Implementation

### The BioXM rationale

BioXM has been developed around the concept of object-oriented semantic integration. In this concept semantically identical objects, which represent information about the same real world object, and the meanings of associations between these objects are identified and mapped based on data and descriptive metainformation [27]. In the life sciences this mostly concerns the mapping of biological entities and descriptive data from literature and databases to common instances of objects like *genes, phenotypes or patients*., Associations between the entities are mapped as relations (e.g. *compound*X *inhibits protein*A) and object - relation information is contextually structured (e.g. *gene*B *expressed in tissue*Z *at time*T *after application of compound*X). Based on objects as nodes (in BioXM called "elements") and relations as edges a "semantic network" which provides semantic information about the connection between participating object instances can be generated.

**Figure 1:**
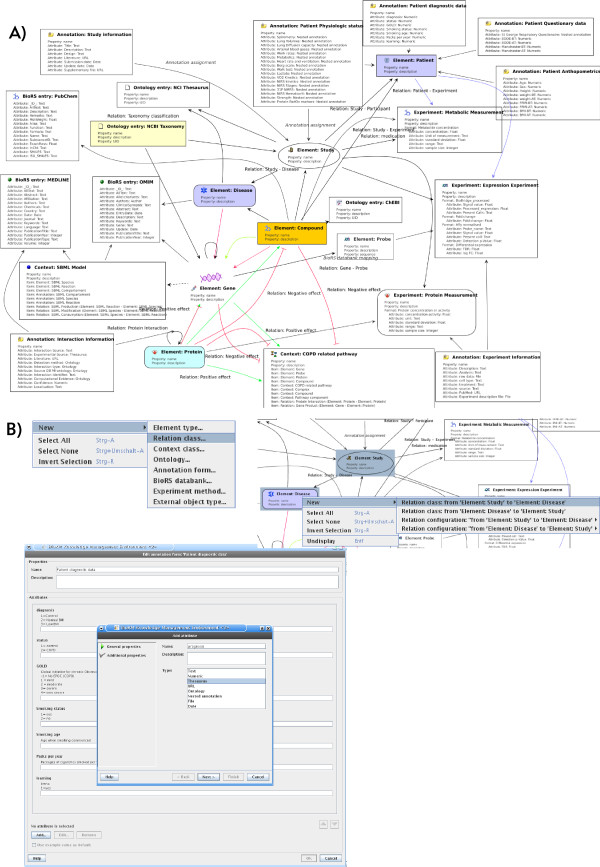
**BioBridge specific BioXM data model**. Partial visualisation of the BioBridge BioXM data model. A) Only the central objects and relations of the data model, such as "Gene", "Protein", "Expression Experiment" or "COPD pathway" are displayed. B) Data model configuration using the data model viewer context menu. New semantic objects are generated and edited directly within the graph viewer.

To enable the extraction of knowledge from integrated information the definition of a protein complex through a series of associations (i.e. "Protein A *participates_in_complex *Complex B") should be supplemented by evidence *why, when *and *where *Protein A participates in Complex B and how we know about it. In BioXM any associated information not represented directly as object or relation can be stored as "annotation" or as a special, performance optimised type "experiment" for high-throughput data. Such evidence may constrain the association to certain conditions e.g. "ProteinA *interacts with *ProteinB during cell cycle phase M". Sub-sections of the semantic network which are true only in specific conditions are modelled as "context". These elementary concepts, "elements", "relations", "annotations" and "contexts" can be defined freely or use existing structured, controlled vocabularies (ontologies). Due to this flexibility when configuring the data model, it becomes possible to formalise huge networks of knowledge (see Figure 1 and Data model configuration) even when concepts as diverse as molecular processes, disease phenotypes or clinical information about patients are concerned. These *complex semantic networks of relationships *allow one to detect connections, extract patterns and answer complex questions like: "Which drugs are known to interact with the over-expressed genes of patient A and have been shown to influence the patients cancer type in a clinical trial?" or "retrieve all inflammation related human genes which interact (physically or functionally) with the metabolic enzymes in skeletal muscle which are affected by COPD".

**Figure 2:**
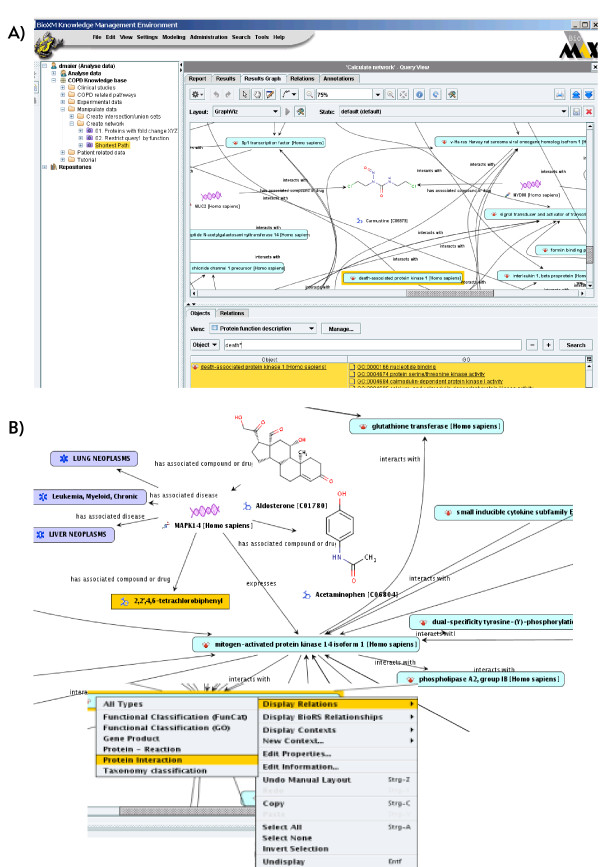
**Querying and visualising the knowledge network**. Visualisation of the knowledge network retrieved by querying for all genes simultaneously showing fold change <-1.0, >1.0 in a COPD expression experiment set, associated with functional information inferred by GO "signal transduction" and connected to each other using the "shortest path" network search. A) Provides an overview of the full retrieved network. B) Focuses on a specific part of the interaction network and the context menu to add gene - disease and gene - compound information for the MAPK14 gene.

**Figure 3:**
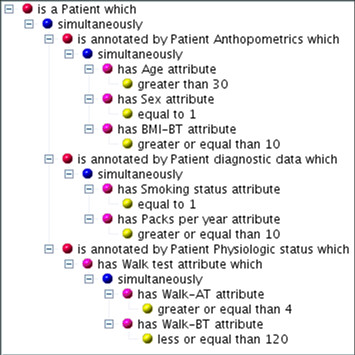
**Natural language like query wizard**. Formulating a natural language query and following the query building steps in the wizard provides a simple way to generate complex queries which can be saved as a smart folder for re-use and other users. Query: "Find all Patients which simultaneously have anthropometric attributes **Age >30**, **Sex male **and **BMI-BeforeTraining >= 10**, are diagnosed with **Smoking status = 1 **and **Packs per >= 10 **and whose Physiology has been measured with **Walk-AfterTraining >= 120 **and **Walk-BeforeTraining <= 100**."

Strong usability is a pre-requisite for user acceptance of any knowledge management system and is approached in several ways in BioXM. The data model itself can be generated graphically and, based on project requirements and processes, may start as a simple sketch converted into a concept map. Parts of the concept map may develop into complex ontologies as discussion and collaboration with the user community extend quick ad-hoc sketched concepts iteratively into precisely defined and structured vocabularies. While not providing an environment for formal ontology development and evaluation such as Protege [28], BioXM allows ontologies to be used for knowledge representation and supports importing, integrating, editing and exporting ontologies. To optimise performance, storage and maintenance resources, data can be managed alternatively within an underlying relational database or be seamlessly integrated from external data sources (i.e. from a users point of view no difference is made between internal and external data). This combines the advantages of a data-warehouse-based deep integration with the low maintenance costs of a federated environment. External information can be incorporated by using the embedded BioRS™Integration and Retrieval System [29] or by directly accessing external objects from e.g. a relational database, a web service or a software application. Integration of the external resource is achieved by XML based registration of the application interface into BioXM. The registration must contain information about the actual programmatic access to the application and the attributes made available. Registered objects become directly available within the graphical data model viewer where they can be mapped in the same way as internal objects (see Data model configuration). External information can serve either as "virtual" semantic objects, or as "read-only" annotation of semantic objects. Read-only annotations add information to any object, relation or other annotation; the information remains within the external data source and is read by the BioXM system on-the-fly but not modified. "Virtual semantic objects" can be organized in the project tree, can become part of a network, and can be annotated by the user like any other object. However, in contrast to BioXM internal objects they can not be changed e.g. renamed and the available search functions and performance depend on the implemented interface and features of the external source. Any change in content in the original data source will become visible in BioXM immediately while full control of the business logic of publishing the objects and associated information remains with the external source. To access the data BioXM provides visual browsing of the knowledge network (Figure 2) as well as "natural language like" data mapping and query wizards (Figure 3) which automatically adapt to the changes of a given data model. The system thus enables scientists to create knowledge networks with flexible workflows for handling data and information types, including annotation and ontologies. In this way research projects can be modelled and extended dynamically to ultimately gain an adequate understanding of whole biological systems. As we are aware of the many issues associated with metainformation based identification of semantically identical objects we do not aim to provide an overall algorithmic solution to these but rather a framework which supports different mapping methods (see Populating the data model) and solves the organisation and use of the resulting semantic network.

### Software Implementation

Details about the technical implementation of the BioXM software have been published elsewhere [30] and are only briefly summarised here.

**Figure 4:**
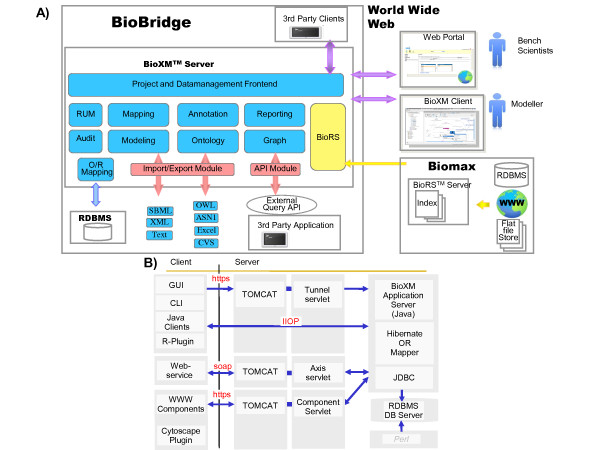
**BioXM architectural concept**. A) The BioXM Java client-server application implements a modular architectural concept on the server side. Tasks such as resource and user management, reporting or graph layouting are implemented as individual modules. B) Inter-module communication and interaction with external applications, web services, browser and Java clients is based on standard protocols.

BioXM is implemented as a platform-independent Java client-server application. The client Java application downloads as a Webstart package and can be started from any Browser (requires pre-installed Java 6.0.4 or newer). The server application has a modular architecture (see Figure 4). The resource and user management module allows control and restriction of user rights to view or modify certain data or parts of the data model. A complete audit trail for all entities and their relations is logged and supports secure data management. The application uses a relational database management system as backend (currently supports MySQL and Oracle). A Hibernate layer is employed for object-relation modelling. Therefore the database scheme is generic, containing the information about the object oriented data model as content. The command line and the SOAP web service application-programming interfaces (API) allow to integrate BioXM into larger bioinformatic infrastructures. Based on these APIs external applications such as BLAST [31] or network search algorithms are integrated. A plugin for the R statistics language http://www.r-project.org/ allows to access BioXM data directly from within the R interface and also to integrate any R method transparently as native BioXM views or analyses.

## Results

The EU FP6 BioBridge Systems Medicine project http://www.biobridge.eu focused on the integration of genomics and chronic disease phenotype data with modelling and simulation tools for clinicians to support understanding, diagnosis and therapy of chronic diseases. We have configured and extended the generic BioXM knowledge management environment to create the knowledge base for this translational system biology approach, focusing on chronic obstructive pulmonary disease (COPD) as an initial use case.

### Data model configuration

In general within BioXM a particular scientific area of interest is semantically modelled as a network of related elements (see Table 1 for a list of fundamental semantic concepts available in BioXM). While there is some agreement throughout the life sciences regarding a number of semantic objects such as *gene *or *phenotype *the different communities such as clinical research, virology, plant research or synthetic biology differ on the concepts and definitions they use. Not only will a plant related knowledge base require the object *plant *instead of *patient*, a *vector *in virology might describe an infectious agent while in synthetic biology it will more likely define a DNA expression shuttle. Different ontology development initiatives (e.g. http://www.obofoundry.org/, http://bioportal.bioontology.org/) try to develop a consensus on these issues but currently no overarching "life science" data model can be defined. Therefore one of the main features of BioXM is the ability to dynamically create a data model specific to the project for which it is used and, while a project develops, easily adapt the data model based on the consensus between the project stakeholders.

**Table 1:** Fundamental semantic objects

Semantic object	Description	Example
Element	Represents a basic unit of a knowledge model	"Gene" element type can be used to create the "STAT3" gene element "Disease term" element type can be used to create the "pancreatic tumor" disease term element

Relation	Describes a relationship between semantic objects	"Gene-disease" relation class can be used to create the "STAT3 is associated with disease pancreatic tumor" relation

Annotation	Extends the properties of a semantic object by a set of attributes	Gene report Patient record Protein entry Literature abstract Experimental data (evidence)

Experiment	Performance optimized extension of an element by a set of attributes	Expression data Metabolomics data Proteomics data

Ontology	Classifies semantic objects according to a defined hierarchical nomenclature of concepts	"3.2.2.21 DNA-3-methaladenine glycosidase II" entry is part of the "EC numbers" ontology Gene Ontology to classify biological function NCI Thesaurus of disease terms taxonomy

Context	Represents sets of semantic objects	Metabolic pathways Protein complexes A disease process or pattern

Database/external object	A basic unit of a knowledge model populated from an external application/database	dbSNP Sequence Variant Genome feature

The fundamental semantic objects used in BioXM which allow a descriptive model of the world to be formulated and data resources to be related to that model. The semantic objects allow an extendable model to be defined from a set of well-defined building blocks (adapted from [30]).

Within BioBridge we had initial discussions between the project partners (clinicians, experimentalists and modellers) about the kind of knowledge that needed to be represented (see Table 2 for the resulting list) as well as how this representation should be conceptualised. This is a typical first step for a knowledge management project which usually provides only an initial, rough idea of the final goal that will need many iterations to reach. Setting-up and changing the data model is done directly within the data model graph (Figure 1) using a context menu to create and edit the fundamental semantic objects (Figure 1B). From the "new" function the basic semantic concepts (element, relation, annotation, experiment, context, ontology, external object) become available to generate for example an element *gene*. By selecting existing object definitions they can be edited, connected by new or existing relations, assigned an annotation or assigned into a context. We created elements such as "gene", "protein", "patient", "compound" and a total of 15 element types sufficed to describe the clinical and biological knowledge relevant to chronic disease (see additional file 1 for a full data model XML export). Information about the interconnection of entities is captured in relations such as "is medicated by" for the compound a patient receives as medication or "regulates" for a protein that regulates expression of a gene. The initial data model was configured within one week and subsequently many iterations extended and adapted the model while the knowledge base was already being populated and in productive use. In total within the current BioBridge COPD knowledge base we used 82 relation types to capture the details of semantic connections between element types, ontology terms, experiments or sub-networks. To describe assemblies of entities or relations with some common feature e.g. a signalling pathway we defined sub-networks as *contexts*. 16 types of contexts where used to capture for example SBML-based simulation models [32], KEGG pathways [33] or inflammatory processes involved in COPD derived by literature mining. All "semantic concepts" (such as *elements*, *relations*, *contexts *or *ontology *) can be associated by annotations with information such as age, weight and gender for a patient, function for a gene or experimental evidence for a protein-protein interaction. Annotations are based on freely definable forms and support hierarchical organisation of information (nested annotation forms). Multiple semantic objects can share annotation to imply relationships. Within BioBridge we defined 61 annotation forms with 892 attributes to provide for example, electronic case report forms for anthropometric, diagnostic, physiologic and questionnaire data. Experimental data is seen conceptually as special, performance optimised annotation. We defined seven experiment formats covering data types such as transcription, metabolite or enzyme kinetics. BioXM supports the conceptualisation of entire areas of interest by using ontologies, which can be used to infer facts and construct abstract queries. 19 different pre-existing ontologies such as GO or the NCI-thesaurus were integrated into the BioBridge data model. The graphical data model builder and the context menu used for the configuration of the BioBridge specific data model is shown in Figure 1. Within BioBridge we focus on the configuration of a data model suited to knowledge, clinical and experimental date around COPD, other instances of BioXM have been configured to cover different diseases or indeed fields of life science research such as enzyme biotechnology or synthetic biology, underscoring the general applicability of the semantic data model configuration process.

**Table 2:** COPD specific knowledge base

Source database	Information type	Current statistics	Level of curation	Updates/Version
**BIND**	Protein interaction Molecular complexes Pathways	6256 Interactions	High throughput data submission and manually curated from literature	last public version 20.3.07

**BioGrid**	Protein interaction	19 707 interactions	Manually curated from literature Different evidence codes	updated monthly

**BRENDA**	Enzyme kinetics	4 729	Manually curated from literature	updated monthly

**ChEBI**	Compound information	15 367	Curated from different data sources	updated weekly

**Comparative** **Toxicogenomics** **Database** (**CTD**)	Compound-gene, Compound-disease and Gene-disease relationships	259 898 relations	Manually curated from literature	updated monthly

**EntrezGene**	Gene functional information	80 793 human, mouse and rat genes	Curated information integrated from different databases, based on RefSeq genomes	updated weekly

**Enzyme**	Enzyme related functional information	4 833	Manually curated from literature	updated weekly

**GEO**	Functional genomics data (expression, ChIP-chip etc.)	>400 000 individual experiments	User submission	updated weekly

**IntAct**	Protein interaction	21 584 binary interactions	Literature curation User submission	updated weekly

**KEGG**	Pathways	418 pathways	Manually curated from literature	updated monthly

**LIGAND**	Compound information	15 185	Manually curated from the published literature	updated monthly

**MIPS** **Mammalian**	Protein Interaction	410 interactions	Manually curated from literature	current release 31.10.07

**OMIM**	Gene - disease relations	20 823	Curated from literature	updated weekly

**Pfam**	Protein family information	10 340 families	Manually curated from sequence alignments	23.0

**ProLinks**	Interaction	>1.4 million	Automatic inference	last release 18.10.04

**PubChem**	Compound information	>26 million	Automatic collection	updated weekly

**PubMed**	Literature abstracts	>19 million	Automatic collection with manual curation	updated weekly

**Reactome**	Interactions and pathways	>600 pathways, >24 000 Inteactions	Manual curation	updated monthly

**RefSeq**	DNA and protein sequences	>11 million	Automatic processing and manual curation	updated weekly

**Unigene**	Transcript sequences	>2 million	User submission followed by automatic clustering	updated monthly

**UniProt**	Protein sequences	>10 million	Automatic processing, Swissprot subsection manual curation	updated bi-weekly

Public data integrated into BioXM in the current version of the BioBridge COPD knowledge base.

### Populating the data model

The model described above enabled us to semantically integrate existing public databases and information derived from the literature with clinical and experimental data created during the BioBridge project.

**Figure 5:**
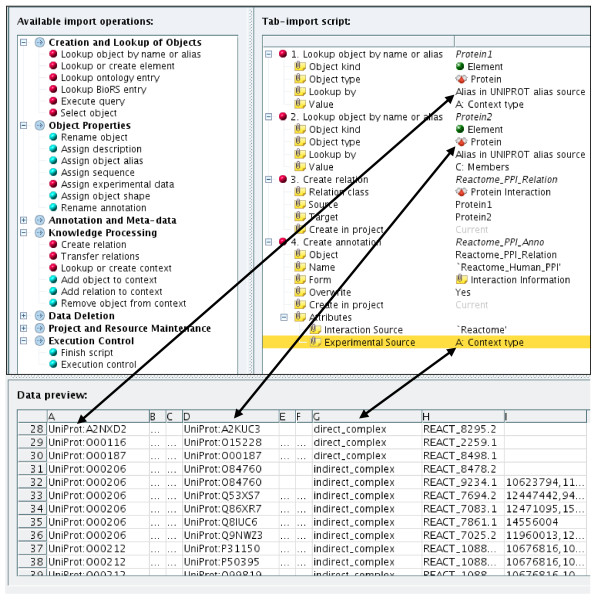
**Populating the data model**. Based on the given data model, the import wizard provides the selection of available import operations in the left frame. These are moved by drag-and-drop into the right frame where they form the import script which provides the mapping information between a data source and the data model. Here two elements from the data source are defined as type "Protein" and are referenced by their UniProt IDs. The relation between the two proteins is a "Protein interaction" from the Reactome data source and the associated evidence is stored as annotation.

To populate the knowledge base with data from public databases with large sizes and regular updates we mainly use virtual objects with a manual mapping of the object concept into the data model. For sources without appropriate interfaces or weak performance and for project internal data and knowledge we manually generated mappings in import-templates. A graphical wizard for import-template generation provides a selection of possible import options and mappings. Only applicable objects of the data model are presented for example after defining an object as *gene *only relations enabled for *gene *are available for the next mapping step. The available selection automatically adapts to any change in the data model configuration. From this selection the import operations are assembled by drag-and-drop to provide the mapping for a given data source forming an import script which can be saved and re-used (see Figure 5). For sources which are imported and provide regular updates a scheduling system is used to define automatic execution of the corresponding access and import methods.

Mapping a resource to the data model requires expertise about the semantic concept of the resource and the configured BioXM data model. To integrate the individual entities of a data source semantically the mapping method for the entities need to be defined. If available, BioXM makes use of namespace based standard identifiers, existing cross-references and ontologies for the population of the data model. In most cases the semantics of a given data source are not (yet) described in machine readable form and the initial mapping template needs to be generated manually. The BioXM core framework is extended with pre-defined semantic mappings currently existing for about 70 public data resources and formats (see additional file 2). In addition text-mining and sequence similarity (BLAST) based mappings are enabled, however users need to be aware of the pitfalls of these methods as no automatic conflict resolution is attempted.

For the COPD knowledge base we use entities, references and ID mappings provided by EntrezGene [34], Genbank [35], RefSeq [36], HGNC [37], ENSEMBL [38], UniProt [39] and EMBL [40] to populate the system with instances of *genes *and *proteins *from human, mouse and rat. Starting with EntrezGene we create gene instances and map entities from the other sources iteratively by reference. For each database the quality of the references to external sources needs to be judged individually and correspondingly be constrained against ambiguous connections. UniProt protein entries for example provide references to DNA databases, with some references pointing to mRNA which allows the corresponding gene to be identified uniquely, and others pointing to contigs and whole chromosomes with multiple gene references. Use of references in this case therefore is constrained to the target entry type "mRNA". A new instance is generated for each database entry from the corresponding organisms which can not be mapped to an existing instance by ID reference; no name based mapping or name conflict resolution is attempted at this stage. As the knowledge base develops iterative rounds of extension occur with additional data sources. Based on non-ambiguous identifiers we map additional information from the sources described below with mappings being extended, removed and remapped during each updating round.

Generating an import template using the import wizard requires no software development knowledge and for many sources only takes minutes (e.g. for protein-protein interaction data which uses UniProt accessions to unambiguously identify the protein entities and the Molecular Interaction Ontology [41] to describe the interaction type and evidence). However, integration can also take up to a week of software development if extensive parsing and transformation of a complex data source such as ENSEMBL is required.

Naming conflicts and lack of descriptive, structured metainformation are the main reasons for the lack of semantic integration in the life sciences, issues that are as much technological as sociological. The use of a structured knowledge management tool within BioBridge ensured all newly produced data makes use of unique identifiers and provides extensive, structured metainformation. This semantic integration and standardisation fostered data exchange as well as social interactions within the project, which are a pre-requisite for translational systems biology projects and their highly diverse multi-subject expert teams. In addition the semantic integration greatly simplifies the future sharing of the produced data as it is immediately available in semantic form.

For the import of data several formats are supported from simple manually mapped delimiter formats such as tab-delimited to XML formats with potentially fully automatic semantic mapping like Pedro [42], SBML or OWL. If machine readable metainformation is provided, such as MIRIAM references in SBML, they are used to automatically map the imported entities to existing instances of semantic objects. In the current version, the knowledge base integrates more than 20 different public databases (see Table 2) representing a total of 80 793 relevant genes (30 246 human, 27 237 mouse and 23 310 rat), 1 307 pathways, 78 528 compounds with related gene/disease information, 1 525 474 protein interactions and the entire Gene Expression Omnibus and PubChem databases resulting in a total of 3 666 313 connections within the knowledge network. In addition two BioBridge specific datasets, 54 inflammation and tissue specific pathways and 122 COPD and exercise specific metabolite and enzyme concentrations and activities were manually curated from the literature within the project. The pathway curation followed a standard text-mining supported process as described for example in [43] while the enzyme concentration and activity curation was fully manual due to the small set of available relevant publications. To our knowledge BioBridge thus provides the first semantically integrated knowledge base of public COPD-specific information. In addition the resource will be continuously extended as more COPD specific data becomes publicly available e.g. the experimental data generated within BioBridge (160 pre- and post-training expression, metabolite and proteomics data sets) will become publicly available as soon as the consortium has generated an initial analysis of the data. Currently the COPD knowledge base contains almost 10 million experimental result data of which almost 6 million come from public data. In other projects we are currently using BioXM with several hundred million data points on networks with tens of million edges and nodes, showing that the approach scales for at least two more orders of magnitude (unpublished data).

### Browse, query and retrieve

**Figure 6:**
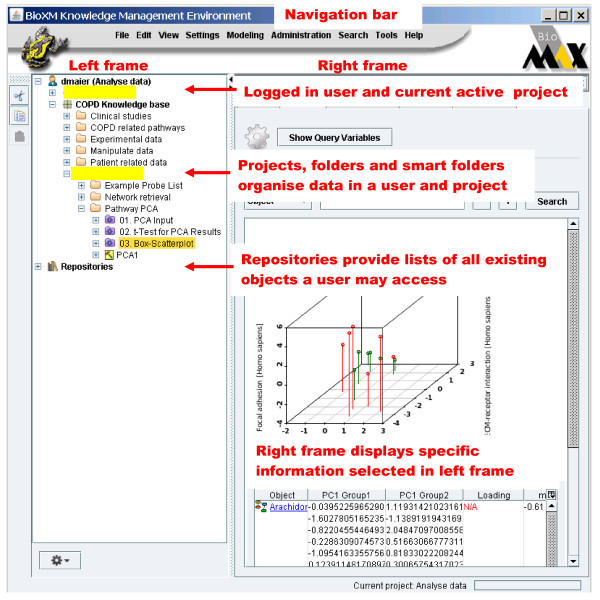
**BioXM graphical user interface overview**. The BioXM graphical user interface (GUI) consists of three frames. A Navigation bar provides the functions for importing, managing, reporting and searching data. A project and repositories frame to the left, allows all data available to a user to be accessed in the repositories section and the data to be organised in a user and project specific way in the projects section. A right frame, is used to display detailed information about any object selected in the left frame.

Here we provide an overview of the available functionality, for a detailed step-by-step tutorial of the knowledge base please see the additional file 3: Step_by_Step_Tutorial_BioXM.pdf. When accessing the BioBridge portal http://www.biobridge.eu/bio/ you will have to register to access the knowledge network (the registration is required to sustain funding support and enable personalisation of the interface, no further use of personal data will be made). Then access the knowledge base by following the BioXM links. The BioXM user interface (Figure 6) provides a visually driven query system with which information can be browsed from a network graph (see Figure 2B), based on pre-defined queries (purple *smart folders *in the navigation tree, see Figure 2A) or by interactive query generation (a detailed application example is described below).

Users visually browse and query the network simply by right-clicking on any focus of interest (e.g. a gene, a patient or a protein-protein interaction) so that associated entities can be added to the existing network visualisation. The corresponding context menu is dynamic, offering all those entities for selection which, based on the data model, are directly associated with the initial focus (i.e. one step in the network). A researcher could, for example, expand from a gene to include its relationships with diseases. From the disease association it may be of interest to identify patients represented in the gene expression database who share that particular diagnosis. Entities distanced by more than one step in the data model can be associated with each other by complex queries which transverse several nodes within the graph and aggregate information to decide whether a connection is valid. These complex queries are transparent to the user who executes them as part of the graphical navigation when asking for "associated objects" (see below for query construction). Graph based navigation will become difficult in terms of visualisation layout and performance beyond several thousand objects.

The intuitive graphic query system therefore is supplemented by a more complex wizard that allows dynamic networks to be created by in depth, structured searches, which combine semantic terms that are dynamically pre-defined by the data model (see Figure 3 for the query wizard and Figure 2A for a resulting network. The additional files 3 and 4 provide details and example data on how to create a query). The query construction is natural language like and thus allows to generate complex searches without knowledge of special query languages such as SQL or SPARQL but some knowledge about the data model must be acquired to work efficiently with the wizard. A search for all patients diagnosed with COPD severity grade above 2 but no cancer which have low body mass index for example would read: "Object to find is a Patient which simultaneously is annotated by Patient diagnostic data which has GOLD attribute greater than 2 and is annotated by Patient Anthropometrics which has BMI-BT attribute less than 18 and never is diagnosed with a NCI Thesaurus entry which is inferred by ontology entry which has name like '*cancer*'". A query can be saved as a "smart folder" or query template for re-use and thus allows experienced users to share their complex queries with less frequent users. For saved queries "Query variables" can be defined so generic smart folder queries can be adapted to specific question. In the example above the actual parameters for COPD severity grade, BMI and diagnosed disease might be set as variables for other users to change. Normal folders (yellow) allow users to organise data manually in their private space by drag-and-drop e.g. to create a permanent list of "favourite genes" or a specific pathway. In contrast the content of "smart folders" is dynamic, as it is actually a query result, which immediately updates whenever changes in the content of the knowledge base occur. Defining a query takes between seconds for simple "search all compounds used as medication" type questions to tens of minutes for complex questions which traverse the full connectivity of the semantic network. In the same way performance of query execution directly depends on the complexity of the query. Queries traversing many connections in the semantic network with entwined constrains may take several minutes to execute while simple queries even with millions of results return within seconds.

**Figure 7:**
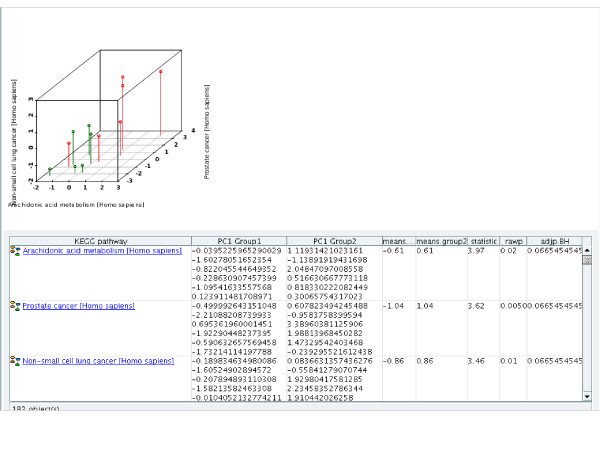
**Report with integrated external application result**. This result view shows the first three significantly different pathways between sedentary and trained healthy people. The 3 D scatterplot on the top visualises the PC1 for each experiment as spot in the 3 D space with the KEGG pathways as dimensions. Green (experiment group 1, here pre-training) and red (experiment group 2, here post-training) spots clearly occupy two different regions of the plot, indicating differences. The significance of the differences is visible in the tabular report where the first column provides the name of the pathway. Column 2 and 3 list the PC1 values for each of the associated experiments in group 1 and 2. Columns 4 and 5 show the overall PC1 mean of the pre- and post-training data. The following columns list the t-, p- and adjusted p-value respectively.

All individual entries, contents of folders and query results are organized in configurable reports presenting the initial element and any desired associated information. To reflect differences in interest and focus for different users multiple reports can be defined for any object for example providing a quick overview of patient laboratory data for the clinician while another patient report provides the expression data for the data analyst. Reports can be exported both in tabular and XML format. Reports can transparently integrate external applications (e.g. the statistical programming environment R) in order to derive visualisation and/or further analysis of the data (Figure 7). The report is based on individual "view items" defined with the same type of wizard as for query generation and import template generation.

Therefore configuring reports does require no software development skill but is based on an understanding of the data model configuration. As with the query and import template wizards, view items are drawn from the data model using functions such as "related object", "assigned annotation" or "query result" which can be further restricted to specific types such as "relation of type protein expression". Configuring a new report on average takes only minutes but, as reports can contain query results, can also take tens of minutes if a new complex query needs to be defined. Reports defined for an object like *gene *can be re-used as "nested reports" wherever a *gene *type object is included as view item in another report allowing complex reports to be assembled from simple units.

Report display performance directly depends on the configuration and takes between seconds and several minutes. Simple, fast reports depend on directly related information e.g. a gene report which brings together Sequence Variant, gene-disease and gene-compound information. Complex, slow reports integrate queries to traverse the semantic network and pull together distant information e.g. the medication for the patients for which a given gene was upregulated.

View items can also be used within "information layers" which visualise the information directly on top of a network graph by changing size and colour of the displayed objects. To define the information layer ranges of expected numerical or nominal values in the view item are assigned to colour and size ranges for the graphical object display. In a simple case this is used to display expression data on top of a gene network but based on using query results as view items it can also be used to display the number of publications associated with a gene - phenotype association. Within the graph information layers are executed for every suitable object displayed and depending on the complexity of the defined view item the generation of the overlay can take between seconds and several minutes.

### Application case

The BioBridge knowledge base implementation enables the integrative analysis of clinical data e.g. questionnaires, anthropometric and physiologic data with gene expression and metabolomics data and literature derived molecular knowledge. The knowledge base is currently used by data analysis and modelling groups within BioBridge to extend literature-derived, COPD-specific molecular networks with probabilistic networks derived from expression data (method described in [44]). Output of the probabilistic networks together with expression and metabolomics data is then used to tune mathematical models of the central metabolism ([45]) for COPD specific simulations.

**Figure 8:**
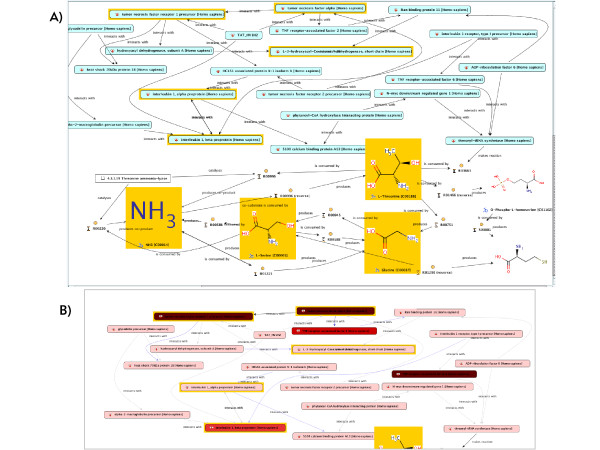
**Network connecting inflammation with central metabolism**. A) Individual nodes (compounds and proteins) are connected based on the shortest path algorithm. The initial compounds and proteins (marked in yellow) have been selected based on their involvement in pathways detected by PCA-pathway analysis and their significantly different concentration/activity in the COPD or training specific literature. Proteins and compounds are connected by following all possible relations within the COPD knowledge network which results in the most parsimonious network revealing a putative mechanistic connection between inflammation related processes and the central metabolism. B) Visualisation of a disease specific pathway association as a proxy for possible protein importance in a disease mechanism. Each protein in the network is queried for the number of associated disease specific pathways manually curated from the literature into the knowledge base. Numbers are visualised from light red (view pathways) to deep red (high number of pathways associated).

As an example use case we briefly describe the initial search for connections between molecular sub-networks affected by exercise, which is generated as a starting point of the BioBridge investigation (for detailed descriptions of this and further use cases see the associated *Step-by-step tutorial*). To this end we searched the expression data in the COPD knowledge base to retrieve studies involving patients diagnosed with diseases affecting muscle tissue or involving "exercise" as treatment. Based on these experiments we used the R interface to conduct a principal component analysis to extract the KEGG pathways most strongly affected by expression changes (R scripts provided in additional files 5 and 6). Interestingly the affected pathways are mainly associated with tissue remodelling and signal transduction pathways. Using the enzyme and compound concentration and kinetic measurements extracted from key manuscripts on muscle dysfunction in COPD and training effects we find that a number of compounds and proteins involved in key pathways with altered expression derived from the principal component analysis show significant changes in concentration/activity in the published literature. Therefore independent support from existing knowledge is gained from the integrated COPD knowledge resource for the statistical analysis results of expression data. For these compounds and proteins we use the network search algorithm to search the entire COPD knowledge network but restrict the allowed connections to human genetic interaction, protein-protein interaction, gene-compound or gene-disease interaction (see Table 2 for the individual sources mapped to each of these relation types). The resulting network connects inflammatory and metabolic processes affected in COPD patients (see Figure 8A and additional file 7: Knowledge_network_table.pdf). Visualising the number of disease specific pathways associated with each of the nodes as information layer in this network immediately provides one aspect of the potential weight of the individual nodes for future investigation (see Figure 8B) with TNF-receptor associated factor 2 and 6 showing the highest relevance in this respect for all newly connected nodes. The definition of the network search can be changed to include multiple additional information types e.g. drug-relations. Weighing of evidence is achieved by specifying penalty weights in the network search parameter set for each type of relation searched. Additionally query results provide further evidence directly visualised as information layer on top of the network graph to change size and colour of the displayed objects. Within the query all attributes available in the knowledge base can be used as described in the previous section, from the number of independent literature occurrences supporting a gene-disease relation to the category of the experimental evidence supporting a protein-protein interaction to derive an informed weighted list of further investigation targets. Different information layers can be defined to overlay different types of information such as quantitative experimental data from gene expression or qualitative text-mining results. The decision about what kind of evidence should be weighted in which way is depending on the individual type of data considered, we do currently not provide automatic scoring or ranking algorithms. However, based on the R plug-in or the API, R-scripts or external algorithms can be integrated to calculate corresponding scores. Due to the fact that information layer can integrate complex queries for many objects this is a performance critical function and may take several minutes to execute.

## Conclusions

While the promises of the Semantic Web continue to creep slowly into existence [46] individual projects need immediate, adaptable solutions which allow project specific knowledge conceptualisations to be set-up with low start-up cost and the flexibility to extend, standardise and exchange their data and knowledge. Using a generic knowledge management framework we were able to configure and populate a productively used, project specific systems biology knowledge base within 6 month with similar, software development based integration projects being reported to take between 2-5 years [13,23]. The COPD knowledge base, set-up as the central knowledge management resource of the BioBridge project, provides a free, comprehensive, easy to use resource for all COPD related clinical research and will be continuously extended aiming to generate the definitive resource on clinical research in COPD. More broadly our configuration based approach to semantic integration is generally applicable to close the knowledge management gap between public and project specific data affecting a large number of current systems biology and high-throughput data dependent clinical research projects. To bridge the gap between the current user interface, tuned to suit experienced, frequent users, and everyday clinical application the BioBridge project developed a simplified web portal interface for a number of use-cases. Based on the feedback from clinical users we recently developed a Foswiki [47] plug in for BioXM which unifies the simple set-up of a Wiki with the knowledge management functions. From this we will develop a browser based portal as the primary access to the COPD knowledge base. Future directions include: support of additional structured languages for import and export such as BioPAX [48]and CellML [49]; development of a workflow framework for data analysis and integration of algorithmic methods for semantic mapping.

## Availability and requirements

The BioBridge COPD knowledge base as a data resource is freely available to academic users (requires pre-installed Java 6.0.4 or newer). Upon request to DM the BioXM software application itself can be made available for academic PhD research projects within the Biomax BioXM PhD collaboration programme.

## Competing interests

DM, WK, MW and SL are employed at Biomax Informatics AG and will therefore be affected by any effect of this publication on the commercial version of the BioXM software. JR, FF, NT, MC, IMM SK and JVF are employed at academic institutes and will therefore be affected by any effect of this publication on their publication records.

## Authors' contributions

DM developed and populated the BioBridge specific data model, provided pre-structured queries and reports and drafted the manuscript. WK devised the BioXM software architecture. MW devised and implemented the BioXM R interface and integration. SGK co-developed the experimental part of the BioBridge data model and provided data analysis. JR conceived the integrative BioBridge approach and provided input to the BioBridge specific data model. FF conceived the data analysis workflow and co-drafted the manuscript. NT implemented the data analysis workflow and co-drafted the manuscript. MC co-developed the BioBridge data model, conceived the literature curation process and co-drafted the manuscript. IMM implemented the literature curation for COPD specific enzyme and compound data. MH implemented the BioBridge portal integration with BioXM. JFV conceived the BioBridge portal architecture. SL devised the BioXM concept and managed its implementation. All authors read and approved the final manuscript.

## Supplementary Material

Additional file 1 **XML export of the full COPD knowledge base data model containing all semantic objects, annotation and experiment definitions**. Click here for file

Additional file 2 **Table of existing mapping scripts and wrappers**. Click here for file

Additional file 3 **Step-by-step tutorial to access and query the COPD specific BioXM instance set-up as part of the BioBridge project**. Click here for file

Additional file 4 **A set of Affymetrix probes used within the Step-by-step tutorial**. Click here for file

Additional file 5 **R script for pathway level principal component analysis of expression data integrated into BioXM and used as part of the analysis described in the Step-by-step tutorial**. Click here for file

Additional file 6 **R script for significance testing of differentially regulated pathways identified by principal component analysis integrated into BioXM and used as part of the analysis described in the Step-by-step tutorial**. Click here for file

Additional file 7 **Analysis of the sub-network connecting inflammation to central metabolism which is derived from the overall COPD knowledge network based on shortest path network search**. Click here for file
