# Supplementary material for: Knowledge management for systems biology a general and visually driven framework applied to translational medicine
Source: BMC Syst Biol. 2011 Mar 5;5:38. doi: 10.1186/1752-0509-5-38 (PMC3060864; doi:10.1186/1752-0509-5-38)
Supplement: Additional file 3 — Step-by-step tutorial to access and query the COPD specific BioXM instance set-up as part of the BioBridge project. [file 1752-0509-5-38-S3.PDF]

Knowledge Management for Systems Biology a general and visually driven framework applied to translational medicine.

Supplementary Material S1, Step-by-step Tutorial

Dieter Maier[1*], Wenzel Kalus[1], Martin Wolff[1], Susana G. Kalko[2], Josep Roca[2], Igor Marin de Mas[4], Nil Turan[3], Marta Cascante[4,] Francesco Falciani[3], Miguel Hernadez[5], Jordi Villà-Freixa[5] and Sascha Losko[1]

[1]Biomax Informatics AG, Robert-Koch-Str. 2, 82152 Martinsried, Germany

[2]Hospital Clinic, IDIBAPS, CIBERES; Universitat de Barcelona, Barcelona, Catalunya, Spain

[3]School of Biosciences and Institute of Biomedical Research (IBR), University of Birmingham, Birmingham, B152TT, UK

[4]Departament de Bioquimica i Biologia Molecular, Facultat de Biologia, Institut de Biomedicina at Universitat de Barcelona IBUB and IDIBAPS-Hospital Clinic, Barcelona, Catalunya, Spain

[5]Computational Biochemistry and Biophysics lab, Research Unit on Biomedical Informatics (GRIB) of IMIM/UPF, Parc de Recerca Biomdica de Barcelona (PRBB); Barcelona, Catalunya, Spain

Contents

1. Introduction 1
2. Scenario 1: Export information from BioXM 2
  2.1. Tutorial Scenario 1 2
3. Scenario 2: Identification and analysis of functional modules involved in response of muscle to exercise. 9
  3.1. Background and Analysis strategy: A study on response to training in healthy individuals 9
    3.1.1. The dataset 9
    3.1.2. Methods 9
  3.2. Tutorial Scenario 2 10
4. Query generation 18
5. Additional information 23
  5.1. BioXM Documentation 23
6. References 23

1. Introduction

BioXM is designed to store, interpret and mine large amounts of biological information. With the purpose of showing the potential of the this application and the knowledge based developed within the EU BioBridge project we have developed a step-by-step tutorial that shows a number of features of BioXM. In addition we demonstrate the use of a functional module analysis technique (Sameith et al., 2008) based on a measure of the overall activity of KEGG pathways (Ogata et al. 1999).
All data, queries and analyses used within the tutorial are available in BioXM as part of the "COPD Knowledge base" read-only project. Users wanting to modify the queries/analyses or interested in creating new queries/analyses will have to do so within the "Analyse data" project for which they have write rights.

The Tutorial covers two scenarios.

- Scenario 1 represents a short general task, it let the user provide a list of genes to create an interaction network integrated from different databases.
- Scenario 2 allows the user to identify significantly different functional pathways between two experimental groups. Extending from this we provide some examples for interactively browsing the genes in the pathway for associated compound and disease information.

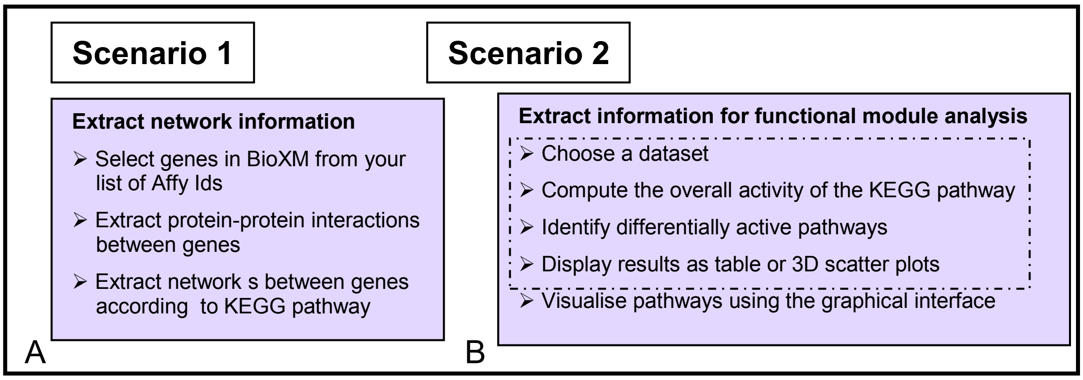

Figure 1: This figure describes the different tasks accomplished in this tutorial. The panel A shows how to extract different types of information. The panel B shows the second scenario where we describe how to extract information and perform a fully integrated analysis using the modularisation approach.

2. Scenario 1: Export information from BioXM

The BioBridge knowledge base offers the ability to dynamically query sub-networks from its huge overall network of connected information and connect the network entities with experimental data. As analysis of high-throughput data depends on the ability to define functional modules it is very important to be able to extract networks of interactions between entities (genes, proteins) integrated from different databases. This information, together with the associated experimental information, can then be used in a large number of data analysis tools. In this scenario, we will show how to extract interactions, as well as gene expression data starting from a list of genes of interest, defined by their Affymetrix probe IDs as provided by the BioXM user. In this example we will extract networks from two different data sources, namely the full set of protein-protein interaction databases integrated in BioXM and the KEGG pathway database respectively. Please note that networks can be extracted based on any combination of data sources integrated into BioXM. The definition of the data source is provided within the query (an example of query construction is given in section 4).

2.1. Tutorial Scenario 1

The following steps will be executed:

- Step 1: Start the BioXM application
- Step 2: Import Affymetrix probe ID list
- Step 3: Extract protein-protein interaction
- Step 4: Extract genes co-occurring in a KEGG pathway

Step 1: Getting started

BioXM is implemented as platform independent Java client-server application and requires a local java installation. Java 1.6.4 or higher is required and can be installed from http://www.java.com/. The application is started by Java Webstart and requires a user registration at http://www.biobridge.eu/bio/.

Open BioXM by accessing the BioBridge portal at http://www.biobridge.eu/bio/. After login/registration click on "BioXM" in the lower right frame of the BioBridge portal, then click on "Search in BioXM".

If you call the application the first time or a new version has been provided at the BioBridge server, the application will download from the server and install itself into your local java environment. A login window will open where you need to provide your user name and password from the registration. Thereafter the BioXM main window opens which is briefly explained in Figure 2.

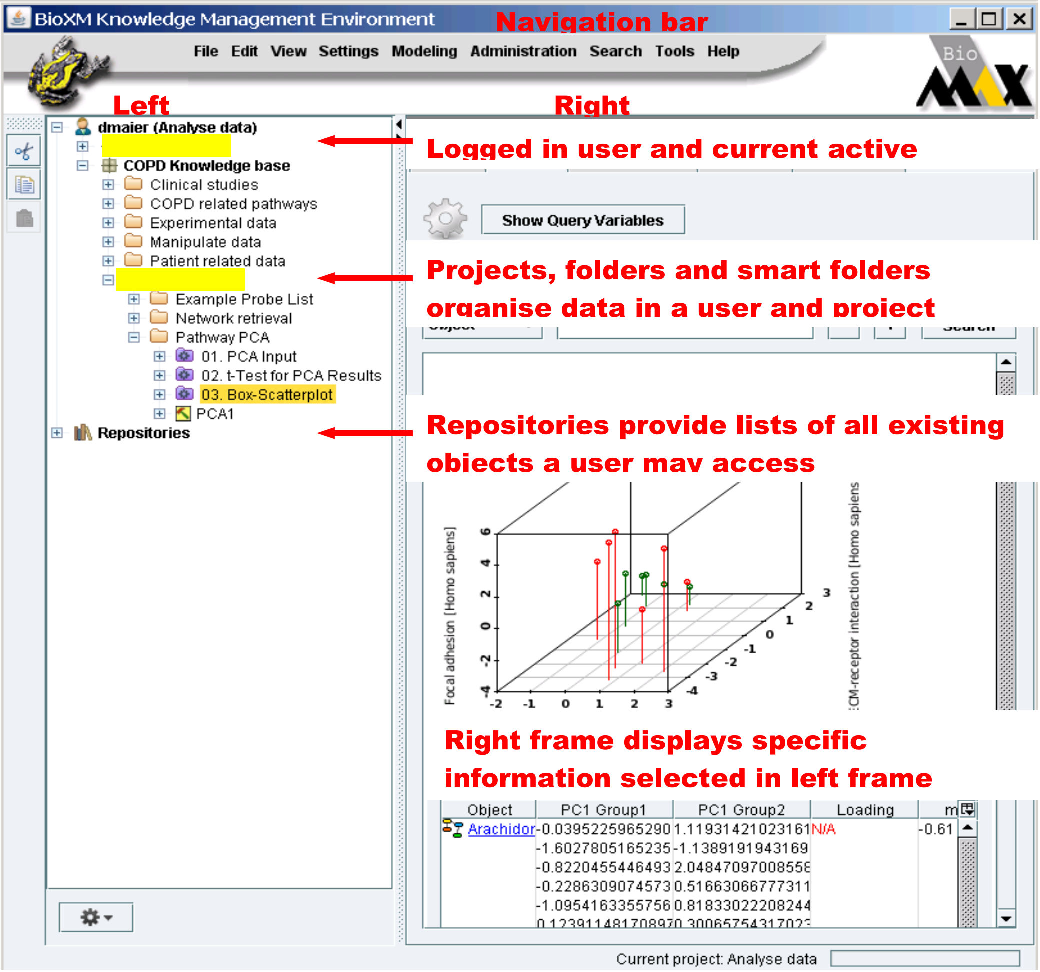

Figure 2: The BioXM GUI consists of three frames, a Navigation bar which provides the functions for importing, managing, reporting and searching data. A Project and Repositories frame to the left, which allows to access all data available to a user in the Repositories section and to organise the data in a user and project specific way in the projects section. And finally a right frame, which is used to display detailed information about any object selected from the left frame.

Step 2: Specify genes of interest by Affymetrix probe ID list (representing genes measured in the expression profiling studies)

We will start by extracting genes represented by a list of 22000 Affymetrix Ids we have selected to be included in the analysis of the first dataset. In this case, the input file with the Affymetrix probe ID list is a text tab delimited file (available in the supplementary material, gse1786.gene.list.txt).

	Action	Background information
i	For your work create yourself a folder to store intermediate data. Right click on the "Analyse data" project in the left frame and select "New folder .." from the context menu. Provide a name for the new folder and click "Ok".	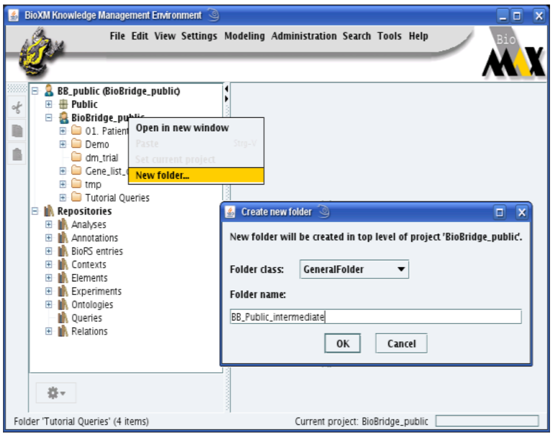
ii	In the navigation bar select "File" -> "Import Table"	Data can be imported into BioXM using different xml formats or as tabular data.<br>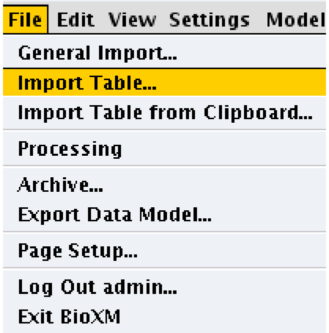
iii	A new window "Import Table Data" will pop up, you can browse the file "S2_gse1786.gene.list.txt" and click "Next".	Data for import has to be accessible on your local client and will be stored within the central BioXM server.
iv	The next screen allows to define the physical layout of your file. In the panel "Header", choose the option "Recognize table header" and click "Next" to continue.	For tabular data the format needs to be specified, first physically e.g. the delimiter (tab, space, ";", etc.).
v	In the next screen, choose "Existing Script" and click "Next" to use a pre-defined import script adding a tabular	Next the semantic format needs to be defined. Data import scripts map objects and their connections in the tabular data to BioXM

	Affymetrix ID list to a BioXM folder.	objects and relations. A number of pre-defined scripts for typical data exist.
vi	Select “Tutorial_List2Folder” from the list of available pre-defined scripts and click “Next”.	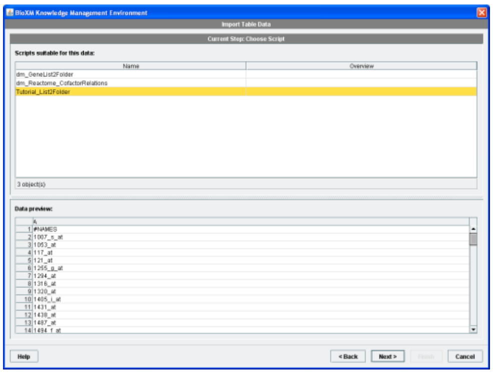
vii	Click on “Add” in the “Folder” Variable of the import script. A new window opens, use the predefined “Tutorial_probe_list” folder or select your own folder from the “Analyse data” project as target for the import data. Click on “Ok” in the folder selection window, then on “Next” in the “Set variable” window.<br>**Note**, while the GUI has been predefined to show only projects relevant to this tutorial, the Folder selection window will show all projects the user has read rights to.	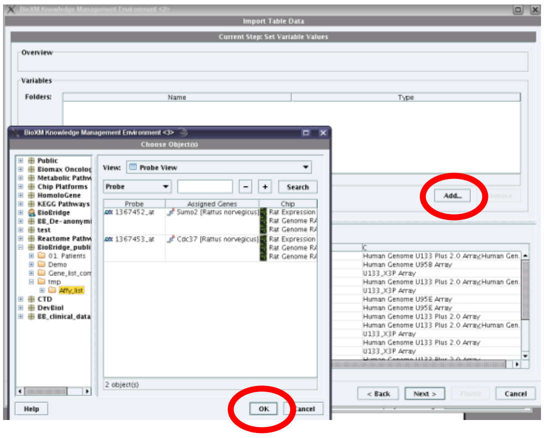
viii	A new screen will appear, choose your processing policy as “Allow partial import” and click “Next” to start the import .	
ix	Once the importing process is completed click “Finish” to exit the wizard.	
x	If you now click on your folder in the left frame you will see its content in the right frame similar to this screenshot	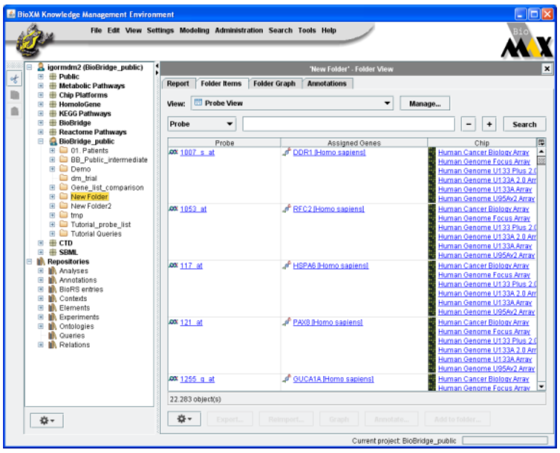

Step 3: Extract protein-protein interactions

	Action	Background information

i	In the left frame open project „COPD Knowledge base”.	The upper part of the left frame contains the project structure. The „COPD Knowledge base” project is read only and contains examples, the “Analyse data” project allows users to generate their own project structure.
ii	In the left frame open folder “Tutorial” by clicking on the (+) sign.	Opening/closing folders by clicking on the (+) and (-) sign respectively will not change the information displayed in the right frame.
iii	In the left frame open folder “Network retrieval”	
iv	Left-click on smart folder “Gene interactions from a probe list”	
v	In the right frame click on “Show Query variables”. Click on add and choose the folder containing the list of Affymetrix probe IDs that you have previously imported during the step 1. Click “Apply” to execute the search.	The result section will provide a list of gene interactions with source gene, target gene and evidence information (Figure 3). The pre-defined folder “Example Probe List” contains 35 Affymetrix probes mapped to genes and yields 34 interactions. **Please note**, using the full list of 22 283 Probes as uploaded in Step 1 the query **will take several minutes** to execute.

vi	In the result table select all (click on any column then use Ctrl-A) and click on "Export" to save the results on your computer.	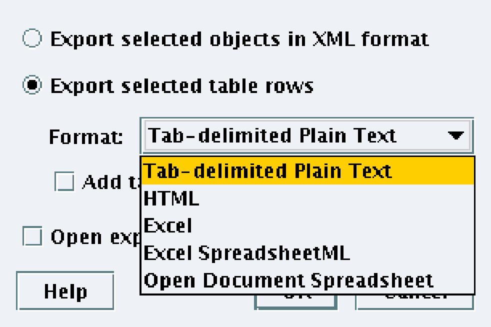 Exports can be stored as xml or in tabular format.
	Click "Close".	

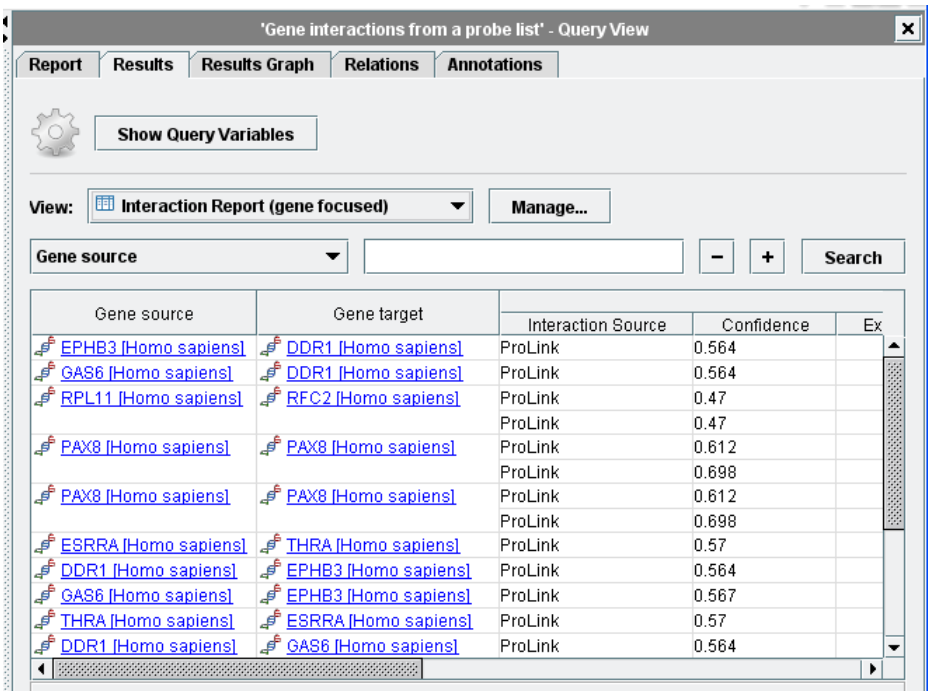

Figure 3: This figure shows the result of the smart folder "Gene interactions from probe list" where the first column "Gene source" is the source of the interaction and 2nd column "Gene target" is the target of the interaction. The 3rd column indicates the source of the interaction and the 4th column shows the confidence of the interaction as provided by the original source database.

Step 4: Extract gene-to-gene interactions as defined by co-occurrence in KEGG pathways

	Action	Background information
i	Left-click on smart folder "extract interactions in KEGG from probes" under "Public/Tutorial/Network retrieval".	
ii	In the right frame click on "Show Query variables". Click on add and choose the folder containing the list of Affymetrix	The result section will provide a list of gene interactions with source gene, target gene and evidence information (Figure 4). The pre-

	probe IDs that you have previously imported during the step 1. Click "Apply" to execute the search.	defined folder "Example Probe List" contains 35 Affymetrix probes mapped to genes and yields 11 interactions within KEGG pathways. **Please note**, using the full list of 22 283 Probes as uploaded in Step 1 the query **will take several minutes** to execute.
iii	In the result table select all (click on any column then use Ctrl-A) and click on "Export" to save the results on your computer.	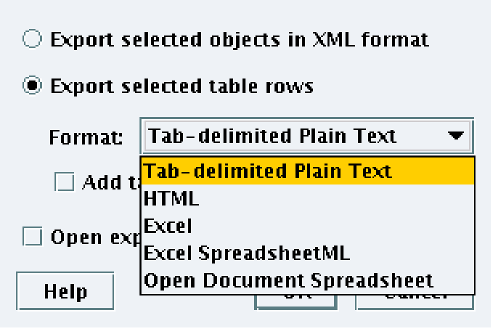 Exports can be stored as xml or in tabular format.
	Click "Close"	

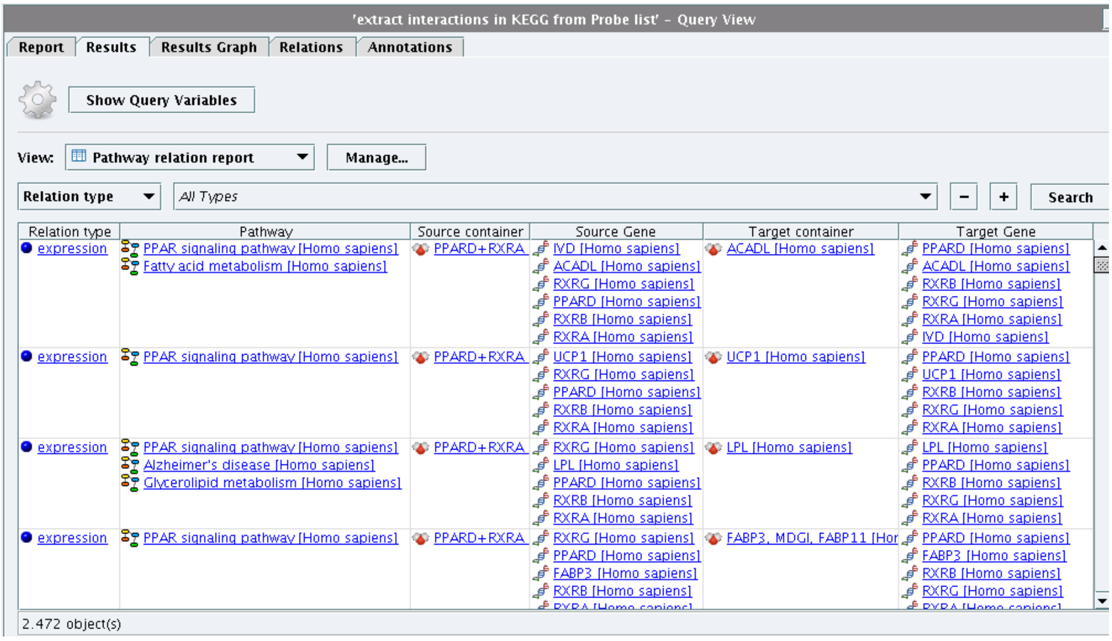

Figure 4: This screenshot shows the result of the smart folder "extract interactions in KEGG from probes". The first column shows the type of the interaction between the source (column 3 labelled "Source container", a container of 1-X genes used by KEGG to describe entities in a pathway) and the target (column 5 labelled "Target container") belonging to the KEGG pathway database (column 2 labelled "Pathway source"). The Genes summarised within the individual container by KEGG are listed as "Source Gene" and "Target Gene" in columns 4 and 6 respectively.

3. Scenario 2: Identification and analysis of functional modules involved in response of muscle to exercise.

3.1. Background and Analysis strategy: A study on response to training in healthy individuals

In this case study, we show the integration of KEGG pathway information with gene expression data applied to the analysis of the transcriptional response of muscle tissues to physical training (Radom-Aizik et al. 2007). We aim to identify KEGG pathways whose activity is modulated in response to training. This modularisation approach has been previously applied in the literature with different strategies using average, principal component analysis (PCA, Sameith et al. 2008) and more complex scores (van Vliet et al. 2007) for summarizing the module activity.

Principal components (PCs) are a good strategy for summarizing the activity of a group of functionally related genes (Sameith et al. 2008). PCA can be used to represent the molecular state of a biological sample in a lower dimensional space while retaining the majority of the information. Briefly, PCs can be considered as linear combinations of the original gene expression measurements (to ensure statistical significance of the PCA we focused on KEGG pathways containing more then 9 genes). The link between expression profiling data and KEGG pathways has been established using a query in BioXM. Once data have been mapped on KEGG pathways, the overall activity of a module has been summarized by its first two principal components.

In order to test whether the overall activity of pathways represented by principal components is different before and after training, we have applied a student t-test comparing two groups of samples and corrected the p-values for multiple testing. Ultimately resulting KEGG pathways of interest are represented graphically in BioXM for further analysis and biological interpretation.

Statistical procedures integrated in BioXM have been implemented in the statistical programming environment R (http://www.r-project.org/).

3.1.1. The dataset

We use a dataset representing the transcriptional profile of muscle biopsies in healthy subjects before and after exercise. This dataset is part of a study developed by Radom-Aizik et al. (2007) and is available from the GEO database (GSE1786). It has 24 samples of needle biopsies from the vastus lateralis of six healthy men and six COPD patients, 67±2.5 year-old males before and after 3 months of training. This dataset has been obtained using the Affymetrix Human Gene chip HG-U133A covering 22283 genes. In our example analysis, we have used only data from healthy individuals for a total of 12 arrays (6 in each group).

3.1.2. Methods

For this analysis, we used the normalized data available in GEO. Figure 5 shows the analysis strategy applied in scenario 1. The overall activity of a KEGG pathway has been defined by its first two components (PCs) computed using the mRNA expression profiles with the *prcomp* method of the R *stats* package. To assess the predictive power of individual modules, we have used these PCs as input of a t-test. The t-test is performed using the R *multtest* package (Pollard et al., 2004) and a Benjamini-

Hochberg (BH) false discovery rate (FDR) correction (Benjamini and Hochberg, 1995) has been applied. The 3-D Scatterplot is produced using the R *scatterplot3d* package.

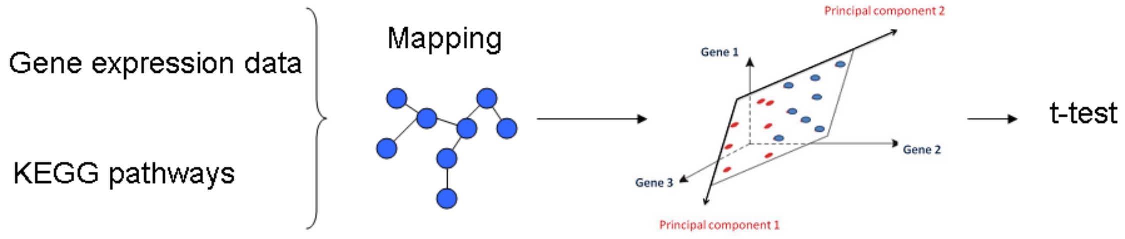

Figure 5: Schematic representation of the modularization analysis.

3.2. Tutorial Scenario 2

The following steps will be executed (assuming the user is already logged into BioXM as described in Scenario 1).:

- Step 1: Execute a query to map experimental data to genes from KEGG pathways
- Step 2: Use the mapped expression data to compute the principal components (PCs) for each KEGG pathway (the principal component analysis (PCA))
- Step 3: Calculate KEGG pathways with associated differential principal components using a t-test.
- Step 4: Visualise PCs for selected KEGG pathways in a 3D-Box-Scatterplot
- Step 5: Interactively browse associated information for genes involved in a given pathway

Step 1: Mapping KEGG pathways and gene list in our data

Queries can be saved as a smart folder within a project folder. The query is performed every time the smart folder is opened, ensuring up-to-date information with no maintenance effort. Query variables allow changing the query e.g. which expression data to include. Here we show how to integrate experimental data with KEGG pathways using a smart folder. A detailed guide on how to construct this specific query "PCA input" is available in section 4. For advanced users willing to develop more complex queries, the BioXM online help has a complete description of the structured query language.

A) Execute the smart folder with predefined variables for which KEGG pathways and experimental data to include:

	Action	Background information
i	In the left frame open folder "Pathway PCA" under Public/Tutorial	
ii	In the left frame right-click on "01. PCA Input" and select "Open in new window" from the context menu.	The smart folder is opened in a new window. The smart folder query is executed to search genes that are part of a KEGG pathway and map the associated expression data chosen by the user (see below in Section 3 for a detailed description of the query). The results of a search within the BioXM knowledge network

		appear as a table of genes with associated information e.g. mapped Affymetrix probe, KEGG pathway, and expression data (**Figure** 6).

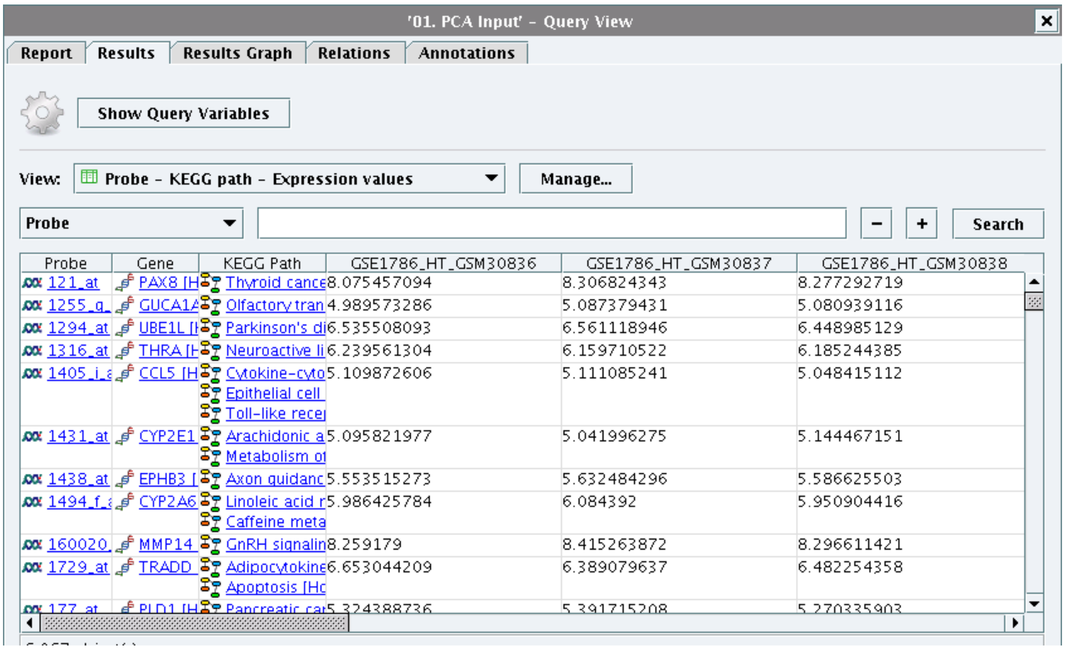

Figure 6: The output of the smart folder "PCA input". The first column "Probe" in the results contains the probes belonging to the selected data, the corresponding gene names are in the second column "Gene". The third column "KEGG path" indicates the KEGG pathway, which the probe belongs to. The rest of the columns show the experimental data associated with each probe.

B) Edit the smart folder variables

	Action	Background information
i	In the query window click on "Show Query variables" on top of the result table.	A new panel opens, which allows to restrict the namespace of searched KEGG pathways or to add experimental data (Figure 7).
ii	In the left frame of the main window open Repositories -> Experiments -> Expression Experiment. Left-click on "Affy normalised". In the right frame left-click on "Experiments"	While some data may be linked into specific projects, to access all available data use the "Repositories" section of the left frame. All experiments available in the "Affy raw" format are listed in the right frame. 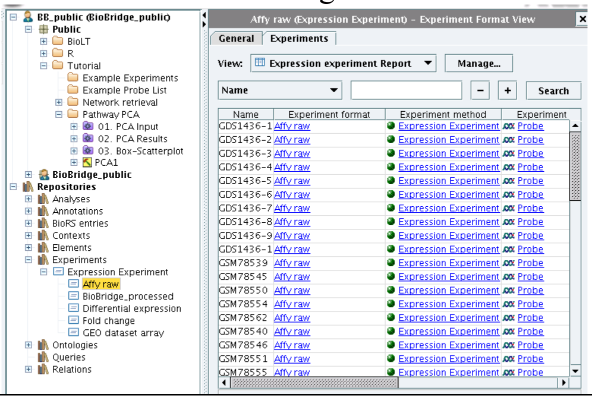
iii	Drag&Drop the experiments you are interested in into the Query Variables	The two groups of experiments will be analysed for differential expression for the list

	"experiment group1" and experiment group2".	of KEGG pathways given.
iv	Run the search "PCA input" by clicking on "Apply"	To change the Query Variables permanently you first have to copy&paste the smart folder into the "Analyse data" project as you need write rights to do so.

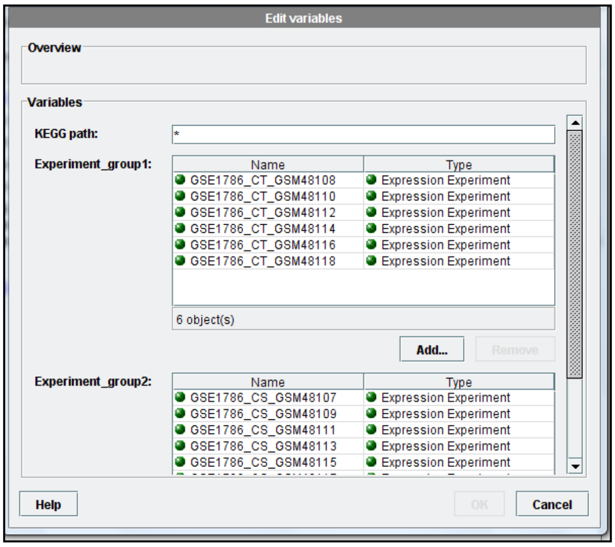

Figure 7: The Query variables for the "PCA input" smart folder, allowing to define the KEGG pathway name (as pattern expression) and the expression data sets (by drag&drop)

Step 2: Computing the overall activity of a KEGG pathway

In this step, the overall activity of KEGG modules is computed using the PCA method as predefined in the BioXM analysis "PCA1". A BioXM analysis allows long running tasks to be executed in the background. The PCA1 analysis transfers the list of KEGG pathways and expression data, as defined in the "PCA Input" smart folder, as input to an R script, which calculates the principal components. The results of the R script are returned to BioXM. Here we describe the steps required to define the PCA1 parameters and start the analysis.

	Action	Background information
i	Left-click on "PCA1" under "Public/ Tutorial/Pathways PCA"	The analysis-associated information will appear in the right frame with 3 tabs. As the analysis has already been run with some data, the "Results" tab visualises the results from the last run as explained in Figure 8.
ii	In the right frame on the tab "Report" click on "Edit parameters".	To start a new analysis, run the "Edit parameter" panel. With your own analysis you could for example provide a different smart folder as input to the analysis.
iii	In the right frame click "Run Analysis".	The analysis run may take several minutes, depending on the number of experimental datasets you specified.

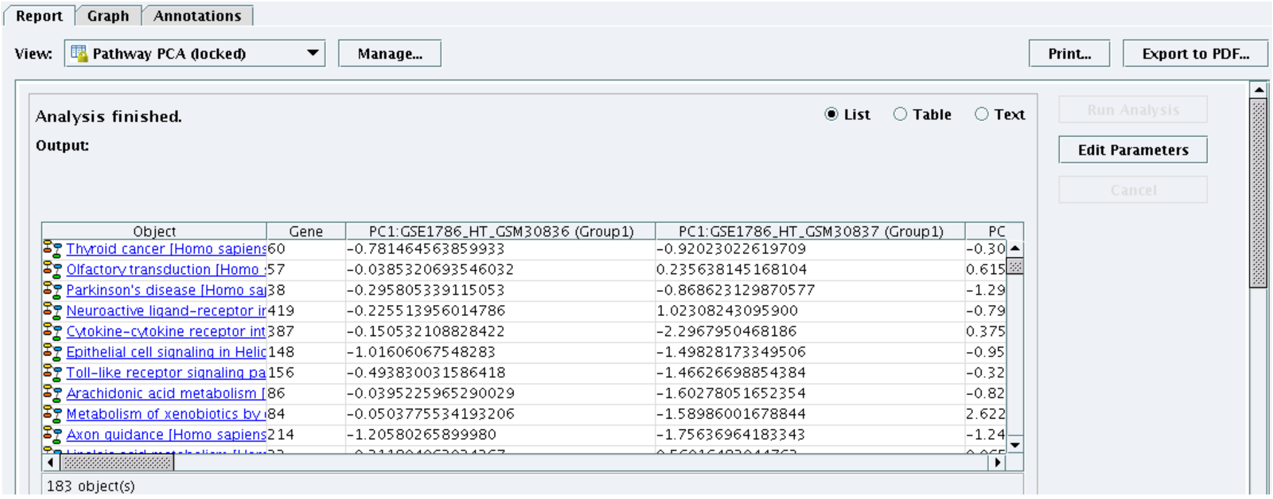

Figure 8: This table shows the result of the PCA analysis. The first column contains the pathway name. Column 2 shows the number of genes associated with the given pathway. Columns 3 and following show the result of the PCA by experiment and PC.

Step 3: Identifying differentially expressed KEGG modules

In this analysis a comparison between the expression state of KEGG modules in sedentary and trained healthy individuals has been performed using a t-test.

The analysis allows to test if the overall activity of the pathway represented by PC1 or PC2 is significantly different in the two experimental groups. Each principal component has been tested independently.

	Action	Background information
i	Left-click on the smart folder “02. t-Test for PCA results” under /Public/Tutorial/ Pathways PCA”	
ii	In the right frame click on the “Results” tab.	A table of KEGG pathways with associated mean PC1 values for each of the experiments and significance p-values (raw and adjusted for multiple testing) are displayed. The PC1s of the three most significant KEGG pathways are visualised as 3D scatterplot on top of the table (Figure 9).

iii	In the right frame, above the 3D scatterplot, click on the "View" drop down menu and select "02. t-test Result PC2".	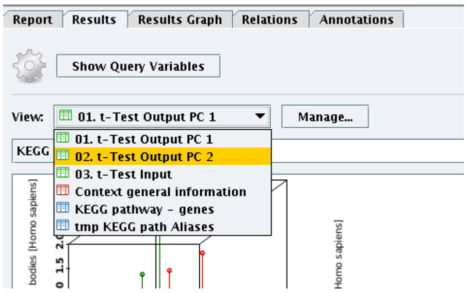<br><br>In BioXM, Views are used to provide custom reports for given objects, to be able to assemble any selection of information associated with the object. Here we have created two separate Views to visualise t-test results for PC1 and PC2.

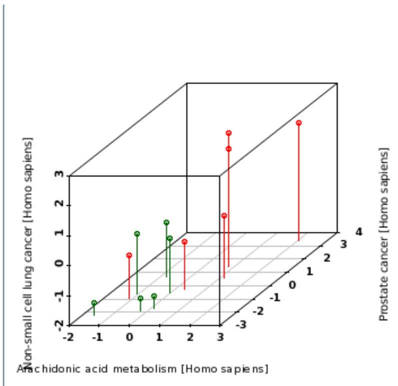

KEGG pathway	PC1 Group1	PC1 Group2	means....	means.group2	statistic	rawp	adjp.BH
Arachidonic acid metabolism [Homo sapiens]	-0.0395225965290029<br>-1.60278051652354<br>-0.822045544649352<br>-0.228630907457399<br>-1.09541633557568<br>0.123911481708971	1.11931421023161<br>-1.13891919431698<br>2.04847097008558<br>0.516630667773118<br>0.818330222082449<br>0.30065754317023	-0.61	0.61	3.97	0.02	0.0665454545
Prostate cancer [Homo sapiens]	-0.499992643151048<br>-2.21088208739933<br>0.695361960001451<br>-1.92290448237395<br>-0.590632657569458<br>-1.73214114197788	0.607823494245488<br>-0.9583758399594<br>3.38960381125906<br>1.98813968450282<br>1.47329542403468<br>-0.239295521612438	-1.04	1.04	3.62	0.0050	0.0665454545
Non-small cell lung cancer [Homo sapiens]	-0.189834634980086<br>-1.60524902894572<br>-0.207894893110308<br>-1.58213582463308<br>-0.0104052132774211	0.0836631357436276<br>-0.55841279070744<br>1.92980417581285<br>2.23458352786344<br>1.910442026258	-0.86	0.86	3.46	0.01	0.0665454545

183 object(s)

Figure 9: This result view shows the first three significantly different pathways between sedentary and trained healthy people. The 3D scatterplot on the top visualises the PC1 for each experiment as spot in the 3D space with the KEGG pathways as dimensions. Green (experiment group 1, here pre-training) and red (experiment group 2, here post-training) spots clearly occupy two different regions of the plot, indicating differences. The significance of the differences is visible in the tabular report where the first column provides the name of the pathway. Column 2 and 3 list the PC1 values for each of the associated experiments in group 1 and 2. Columns 4 and 5 show the overall PC1 mean of the pre- and post-training data. The following columns list the t-, p- and adjusted p-value respectively.

The t-test shows that training is associated to a profound change in the muscle transcriptional activity. Of particular interest is the finding that pathways involved in inflammation, cell-to-cell communication (exemplified by a number of cancer related

pathways), cell remodelling, biosynthesis and metabolism are differentially modulated between the sedentary and trained healthy individuals.

Step 4: Scatter Plots

As alternative view on the analysis it may be useful to generate a 3D scatterplot for a set of user defined pathways instead of the three most significantly different pathways as shown above.

In this example we will visualize the separation between the 12 muscle biopsies as a function of the second components of the pathways "Tight Junction, Gap Junction and ECM receptor. These are among the most differentially modulated pathways in response to training according to our analysis. They are also rather interesting from a biological prospective since they are playing an important role in tissue remodelling.

	Action	Background information
i	In the left frame left-click on the smart folder "03. Box-Scatterplot" under "/Public/Tutorial/ Pathways PCA". Click on the "Result" tab in the right frame.	The 3D scatterplot and t-test result for the 3 currently defined pathways becomes available.
ii	In the right frame click on "Show Query Variables" to edit the names of the KEGG pathways you want to visualise.	Pathway names need to be complete or be extended with a wildcard "*" as BioXM searches for complete matches, not sub-string matches (Figure 10 A).
iii	Click on "Apply"	The 3D scatterplot and t-test for the defined KEGG pathways becomes available (Figure 10 B).

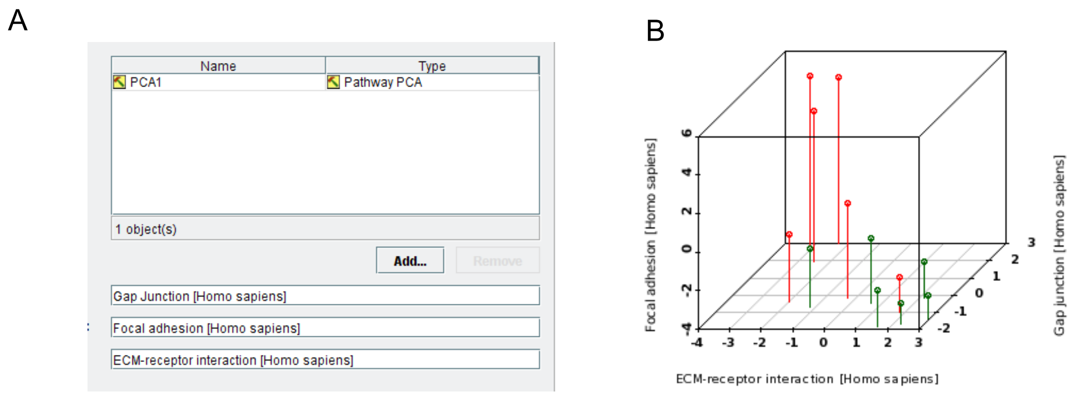

Figure 10: The panel A shows the screenshot on the process of choosing the pathways to plot. The panel B shows the 3D scatterplot, result of the smart folder "Box-Scatterplot".

Step 5: Visually browsing information in BioXM:

A large number of information is integrated in BioXM. This information can be explored using a graphical interface. Moreover, BioXM also offers the possibility to query whether, for example, genes in the pathways have been involved in other forms of disease or are targets of known drugs.

	Action	Background information
i	Click on the “Type II diabetes mellitus [Homo sapiens]” link in the t-test Output PC1 result or search a pathway of interest as described in ii.	A new window will open providing different ways of exploring the selected pathway (Continue at iii).
ii.a	**Alternatively**, in the left frame click on “KEGG pathway” under “Repositories/Contexts/”, then click on the “Context” tab in the right frame.	Within BioXM, pathways are defined as sub-networks or “Context” of the complete knowledge network. Different types of sub-networks are distinguished by their Context class e.g. KEGG pathways, Biochemical pathways, Reactome pathways.
ii.b	In the right frame, above the list of available pathways write in the empty search field “Type II diabetes*” and click on search.	A quick search will filter the result table in the right frame for the search pattern and provide only objects with matching names.
ii.c	Double-click on the “Type II diabetes mellitus [Homo sapiens]” in the result table	A new window will open providing different ways of exploring the selected pathway e.g. accessing the list of objects or relations participating or using a graph display as shown below.
iii	Click on the tab “Context Graph” in the new window.	The pathway will be visualised graphically (Figure 11 A).
iv	To browse gene-compound relations, right click on a component, for example, “IRS1”. A menu will pop up, choose first “Display Context Object” and then “Element: gene”.	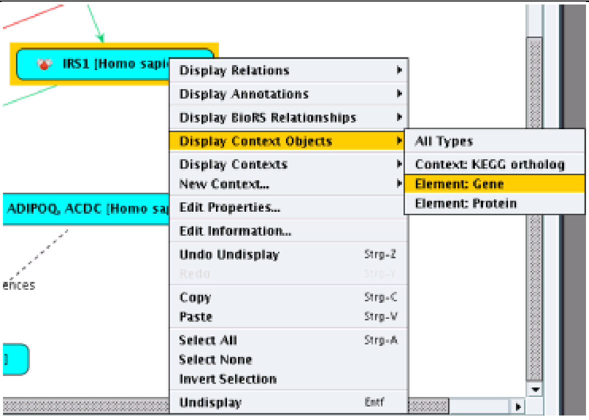
vi	Right-click on the displayed gene “IRS1” and choose “Display Relation” -> “gene-compound relation” from the menu, which will pop up.	As more than 10 compounds are related to this gene, the relations are not displayed directly; instead a selection table will appear which allows the user to mark those compounds you are interested in.

vii	From the pop-up window listing all compounds related to “IRS1” mark the first three compounds (left-click the first, then shift left-click the third) and click “OK”	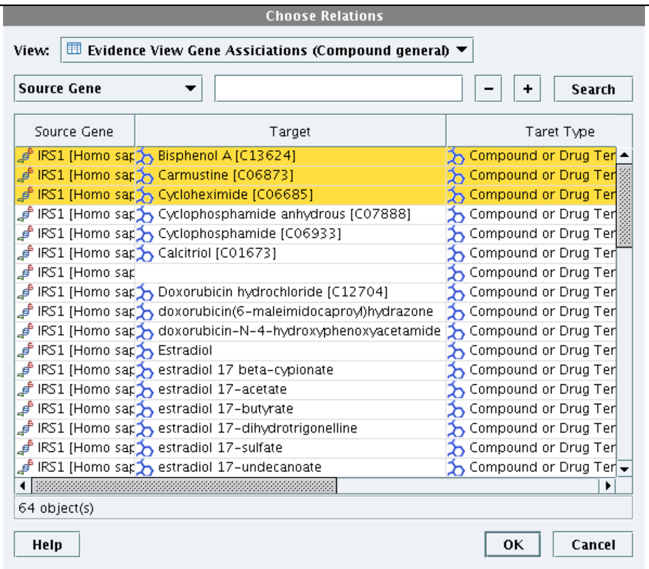<br>The selected compounds are displayed in the graph with their connection to the “IRS1” gene (<br>Figure 11 B).
viii	Double-click on the Relation between “IRS1” and “Carmustine” to view the evidence for this connection	Every Object and Relation in BioXM can be tagged with Annotation e.g. to provide the evidence behind a Relation.
vii	Shift left-click on the compound “Carmustine”, then on the Compound “Bisphenol A” so both are marked. Right-click on any one of these and choose “Find Connections between selected objects” -> “via Element:Gene” from the context menu.	A number of additional genes associated with both compounds are displayed (Figure 11 C).

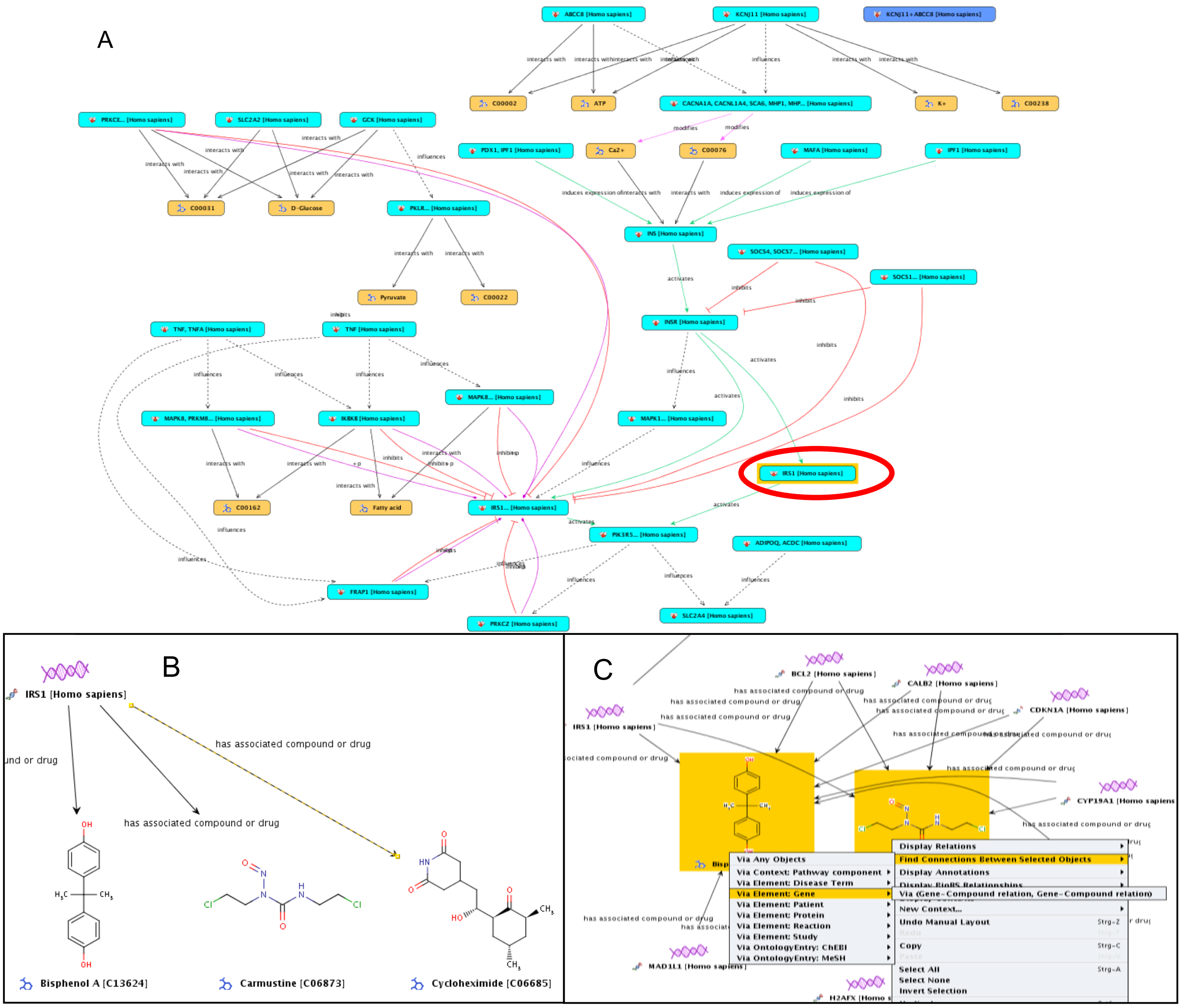

Figure 11: This screenshot shows the graphical visualization of the pathway "Type II diabetes mellitus [Homo sapiens]. The blow-ups provide two results for the visual browsing of the knowledge network, from gene to compound and connecting two compounds within the network.

4. Query generation

1. Description of the query executed within the smart folder "PCA input"

The query "*PCA input*" uses the result of a pre-defined query template called "***retrieve genes in KEGG pathway***". Therefore, we describe these two queries. Before starting to prepare a query it is important to take into account the data model (i.e. the structure of items) in BioXM (see Figure 12). For example in this query, the "Pathway component" is a container used by KEGG to assemble different gene or protein isoforms. So the query needs an intermediate step from gene to Pathway component to pathway. There are also proteins and protein-complexes as additional intermediate steps.

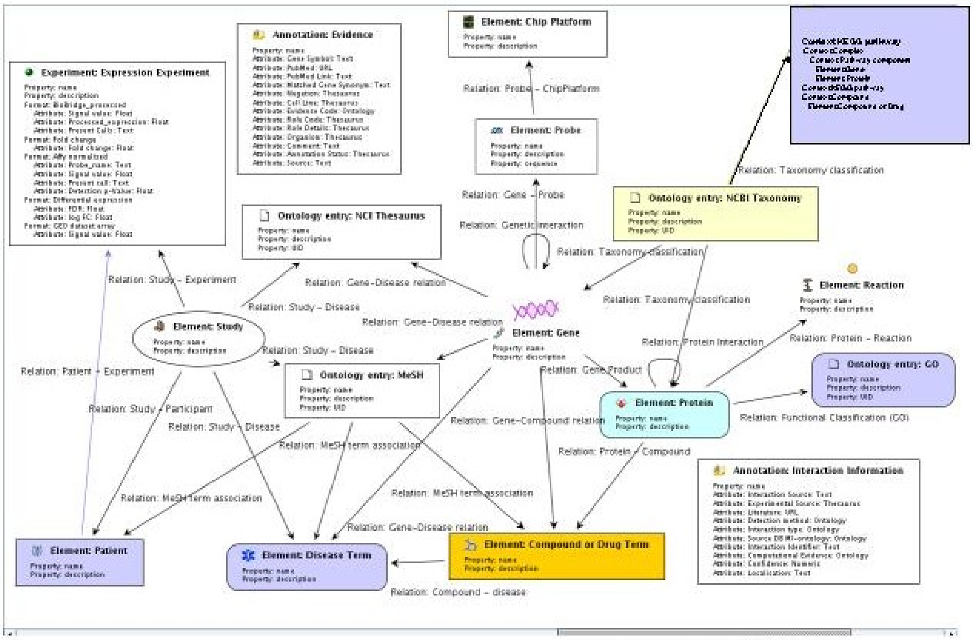

Figure 12: Partial visualisation of the BioBridge BioXM data model. Only the central objects and relations of the data model, such as "Gene", "Protein", "Expression Experiment" or "KEGG pathway" are displayed.

Please note, while all users are able to view the pre-defined smart folders and query templates in the „COPD Knowledge base" project, only users with specific rights are allowed to edit them. Normal users can create and edit new smart folders, query templates and queries within the "Analyse data" project.

To view the query used within "PCA input" right-click on the smart folder in Public/R/Pathways PCA and select "Edit smart folder ..." (Figure 13).

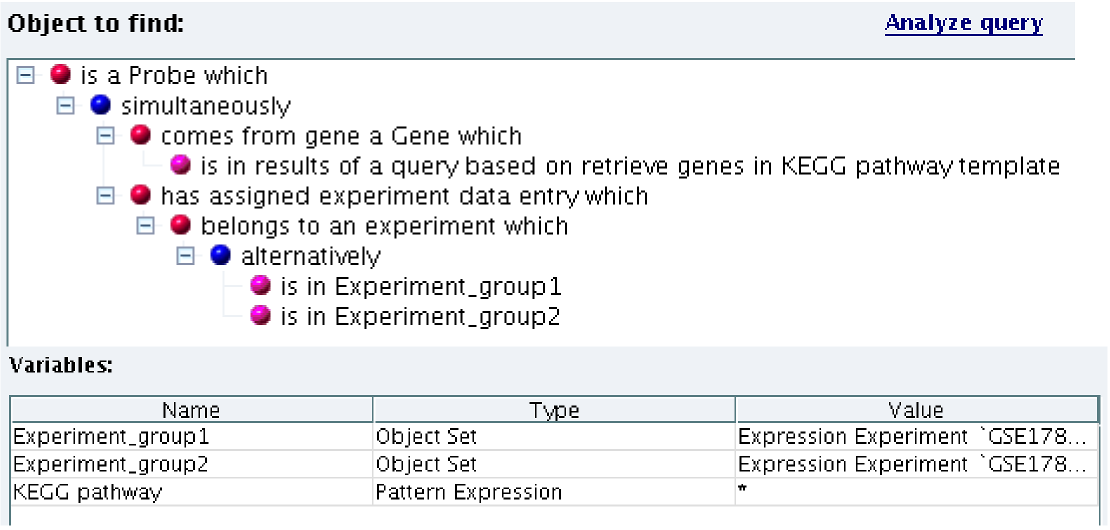

Figure 13: Query used in the smart folder "PCA input"

To view the "retrieve genes in KEGG pathway" query template click on "Search" in the navigation bar and then on "Edit Query Templates ...". In the new window select "retrieve genes in KEGG pathway" and click on "Edit Definition" (Figure 14).

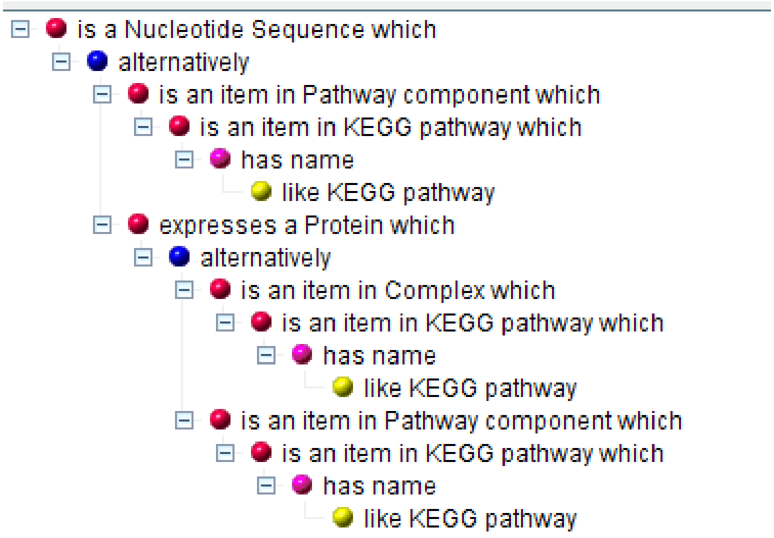

Figure 14: Query Template "retrieve genes in KEGG pathway"

The following schema explains the query “PCA input”:

The query “ **PCA Input** (c.f. Figure 13) searches for all probes which:

A) are associated with a gene which is part of a KEGG pathway
, this part of the Query actually uses an existing
query template "retrieve genes in KEGG pathway" (c.f. Figure 14)

The idea behind **"retrieve genes in KEGG pathway"** is: search for genes which

A) are an item in a Pathway component which
- is an item in a KEGG pathway

OR “ alternatively”

B) express a Protein which
- is an item in a Pathway component which
  - is an item in a KEGG pathway

OR “alternatively”

- is an item in a Complex which
  - is an item in a Pathway component which
    - is an item in a KEGG pathway

AND “simultaneously”

B) have an assigned experimental value, which has been measured within one of the experiments given in the list

A) Experiment_group 1

OR “alternatively”

B) Experiment_group 2

2. Creating a query similar to the "PCA input" smart folder

i. "Search" → "Find by Advanced Query"

ii. A new window will appear, double-click on "is an element" on the left most panel. Then precise the element type "probe" on the right panel. And click on "Apply" on the same panel.

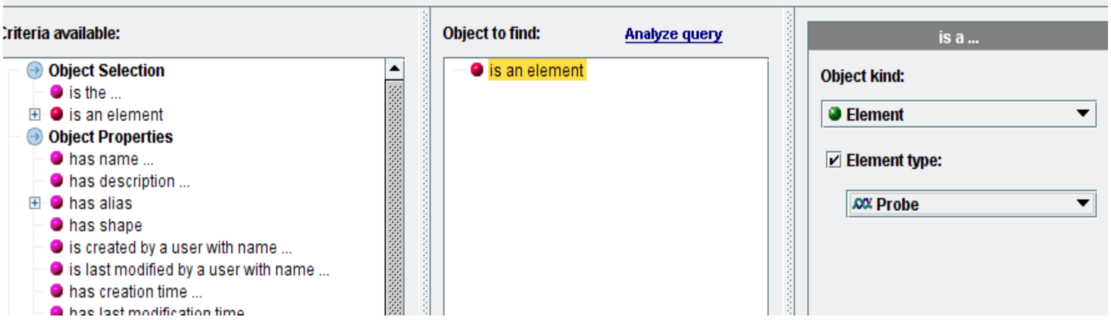

Figure 15: This screenshot shows the query window at the end of the step (ii.)

iii. Double-click on "simultaneously…" under "Logical" on the left panel.

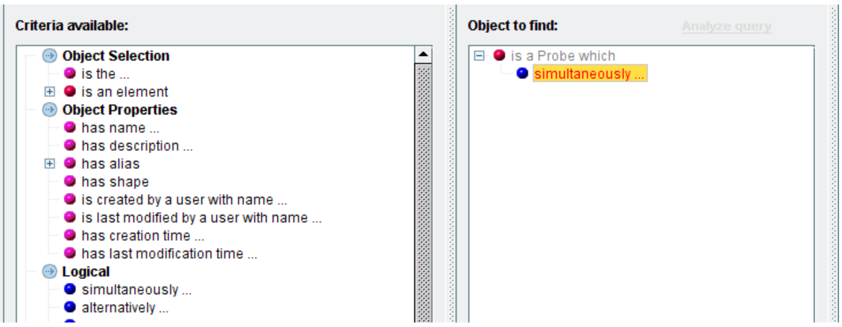

Figure 16: This screenshot shows the query window at the end of the step iii

iv. Double-click on "is in results of a query based … on template" under "Object Association" on the left panel. Then on the right panel, click on "Choose" to choose your query template. A new window will pop up; choose **"retrieve genes in KEGG pathway".** Click "OK". Under "Edit Variable Values" in the right panel provide a value for the KEGG pathway name e.g. "*" to search across all KEGG pathways.

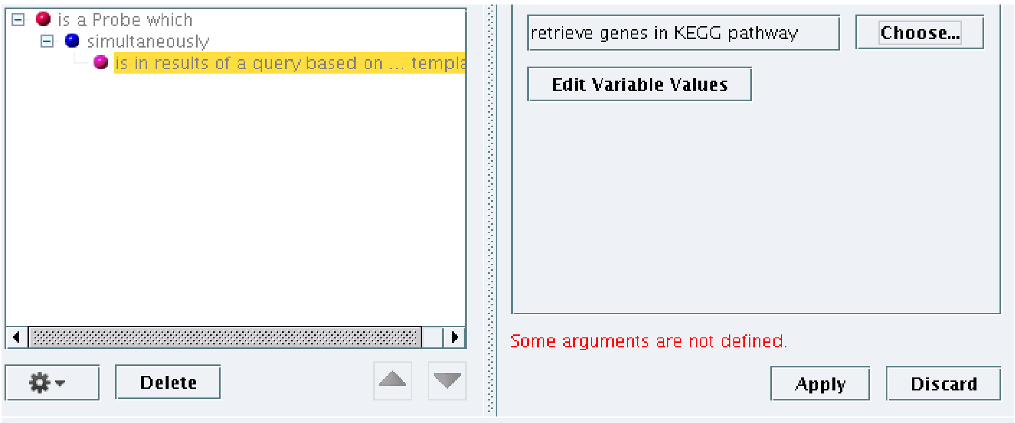

Figure 17: This screenshot shows the query window at the end of the step iv

v. click on “simultaneously” in the middle panel to get back to the object selection on the left panel. Double click on “has assigned experiment data entry which ...” under “Experiment”. Double click on “belongs to an experiment which ...” under “Experiment”. Double click on “alternatively” then on “is the” under “Object selection”. Repeat the last step.

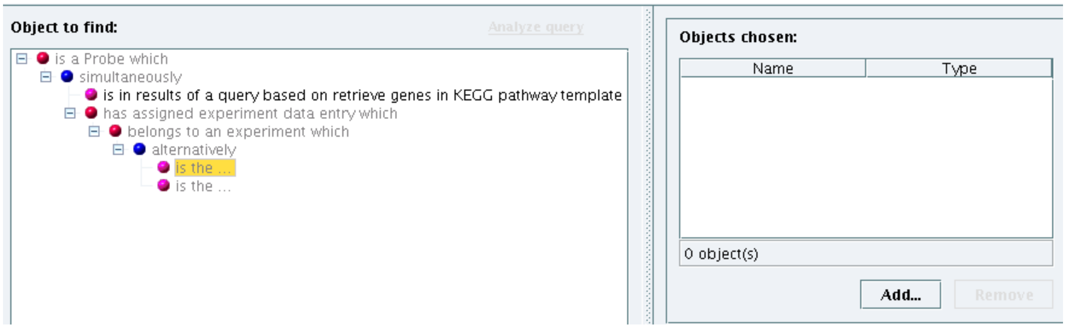

Figure 18: This screenshot shows the query window at the end of the step v

vi. Click on “Add” in the right panel and in the new window select your experiments of interest for group 1 from a given folder. Repeat for group 2.
Note: within this selection box you currently can not select Experiments directly from the repository, you have to move them into a folder first.

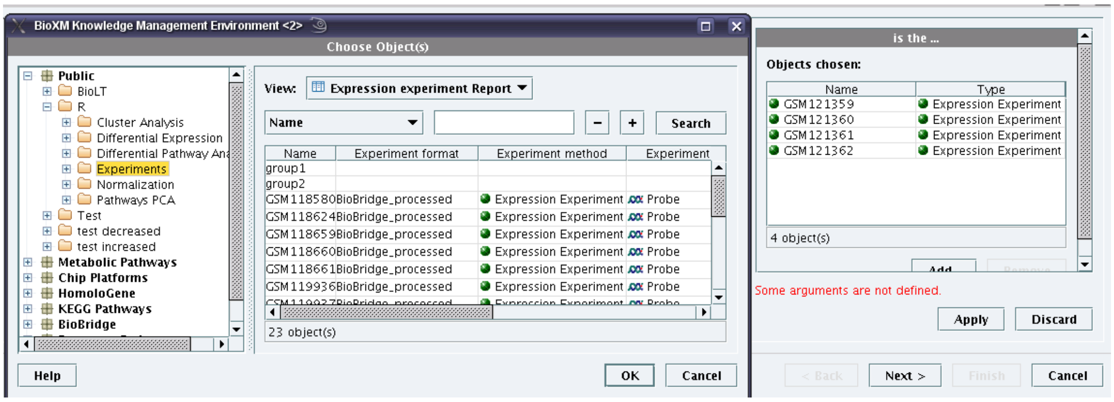

Figure 19: This screenshot shows the query window of the step vi.

vii. Click “Next” on the bottom of the window to execute the Query.

viii. Click on “Next” in the Query result table to save the query as simple query, query template or smart folder. Query templates and smart folders allow to introduce variables instead of e.g. the fixed list of experiments used in the current query.

5. Additional information

5.1. BioXM Documentation

An Online help is available from the "Help" menu in the navigation bar within the BioXM Knowledge Management Environment application. The "Help" section provides also links to an Online Tutorial and further Documentation.

6. References

Benjamini and Y. Hochberg. (1995) Controlling the false discovery rate: a practical and powerful approach to multiple testing. *Journal of the Royal Statistical Society*, Series B, 57:289.

Ogata, H., Goto, S., Sato, K., Fujibuchi, W., Bono, H. and Kanehisa, M. (1999) KEGG: Kyoto Encyclopedia of Genes and Genomes. *Nucleic Acids Res.* 27, 29-34.

Pollard, K.S., Dudoit, S. and van der Laan, M.J., (2004) Multiple Testing Procedures: R multtest Package and Applications to Genomics. *The Berkeley Electronic Press* Paper 164, http://www.bepress.com/ucbbiostat/paper164

Sameith K, Antczak P, Marston E, Turan N, Maier D, Stankovic T, Falciani F: Functional modules integrating essential cellular functions are predictive of the response of leukaemia cells to DNA damage. *Bioinformatics* 2008, **24**:2602-7.

Radom-Aizik, S., Kaminski, N., Hayek, S., Halkin, H., Cooper, D. M., and Ben-Dov, I. (2007). Effects of exercise training on quadriceps muscle gene expression in chronic obstructive pulmonary disease. J. Appl. Physiol 102, 1976-84.

van Vliet, M. H., Klijn, C. N., Wessels, L. F. A. and Reinders, M. J., T. (2007) Module-Based Outcome Prediction Using Breast Cancer Compendia. *PLoS ONE* 2. (DOI10.1371/journal.pone.0001047)
