# Supplementary material for: Knowledge management for systems biology a general and visually driven framework applied to translational medicine
Source: BMC Syst Biol. 2011 Mar 5;5:38. doi: 10.1186/1752-0509-5-38 (PMC3060864; doi:10.1186/1752-0509-5-38)
Supplement: Additional file 7 — Analysis of the sub-network connecting inflammation to central metabolism which is derived from the overall COPD knowledge network based on shortest path network search. [file 1752-0509-5-38-S7.PDF]

Table 1 - Knowledge network

Based on shortest path network analysis the sub-network connecting inflammation to central metabolism is derived from the overall COPD knowledge network.

Interaction Partner 1	Interaction Partner 2	Interaction Source	Confidence	Experimental Source	Detection method	Interaction type	References (PubMed ID)
R00366	NH3 [C00014]	KEGG					
R00365 (reverse)	Glycine [C00037]	KEGG					
R00366 (reverse)	Glycine [C00037]	KEGG					
R00581 (reverse)	L-Serine [C00065]	KEGG					
Ran binding protein 11 [Homo sapiens]	L-3-hydroxyacyl-Coenzyme A dehydrogenase, short chain [Homo sapiens]	ProLink	0.784		RS Rosetta Stone/Gene Fusion		
4.3.1.19 Threonine ammonia-lyase	R00220	KEGG					
NH3 [C00014]	R00220 (reverse)	KEGG					
tumor necrosis factor alpha [Homo sapiens]	tumor necrosis factor receptor 1 precursor [Homo sapiens]	Reactome direct_complex;IntAct;BIND;BioBridge textmining		elisa: enzyme-linked immunosorbent assay	MI:0007 anti tag coimmunoprecipitation	MI:0218 physical interaction	12887920;2848815;14743216;7852363;15355854;11279061;10465784;11704541;11934805;17906365;7852363
tumor necrosis factor receptor 2 precursor [Homo sapiens]	tumor necrosis factor alpha [Homo sapiens]	IntAct			MI:0676 tandem affinity purification	MI:0218 physical interaction	14743216
S00001 (reverse)	O-Phospho-L-homoserine [C01102]	KEGG					
R00588 (reverse)	L-Serine [C00065]	KEGG					
interleukin 1, alpha proprotein [Homo sapiens]	S100 calcium binding protein A13 [Homo sapiens]	BioGrid;BioGrid		Invitro;Affinity Capture-MS			12746488;16189514
L-Threonine [C00188]	R00996	KEGG					
R01221	Glycine [C00037]	KEGG					
TAT_HV1H2	tumor necrosis factor receptor 1 precursor [Homo sapiens]						
Glycine [C00037]	R00588 (reverse)	KEGG					
N-myc downstream regulated gene 1 [Homo sapiens]	threonyl-tRNA synthetase [Homo sapiens]	IntAct			MI:0006 anti bait coimmunoprecipitation	MI:0218 physical interaction	17220478
ADP-ribosylation factor 6 [Homo sapiens]	threonyl-tRNA synthetase [Homo sapiens]	IntAct			MI:0006 anti bait coimmunoprecipitation	MI:0218 physical interaction	17353931
R00220 (reverse)	L-Serine [C00065]	KEGG					
NH3 [C00014]	R01221 (reverse)	KEGG					

interleukin 1, beta proprotein [Homo sapiens]	interleukin 1, alpha proprotein [Homo sapiens]	ProLink	0.663		GC Gene Cluster		
NH3 [C00014]	R01221	KEGG					
phytanoyl-CoA hydroxylase interacting protein [Homo sapiens]	N-myc downstream regulated gene 1 [Homo sapiens]	IntAct			MI:0398 two hybrid pooling approach	MI:0218 physical interaction	16169070
Glycine [C00037]	R03389 (reverse)	KEGG					
R00945 (reverse)	Glycine [C00037]	KEGG					
R00364	NH3 [C00014]	KEGG					
Glycine [C00037]	R00945	KEGG					
L-Threonine [C00188]	R01466 (reverse)	KEGG					
4.3.1.19 Threonine ammonia-lyase	R00996	KEGG					
L-Serine [C00065]	R00945 (reverse)	KEGG					
NH3 [C00014]	R00364 (reverse)	KEGG					
L-Homocysteine [C00155]	S00001 (reverse)	KEGG					
Glycine [C00037]	R00751 (reverse)	KEGG					
hydroxyacyl dehydrogenase, subunit A [Homo sapiens]	L-3-hydroxyacyl-Coenzyme A dehydrogenase, short chain [Homo sapiens]	ProLink;ProLink	0.581;1.0		RS Rosetta Stone/Gene Fusion;PP Phylogenetic Profile		;
R00996 (reverse)	L-Threonine [C00188]	KEGG					
threonyl-tRNA synthetase [Homo sapiens]	R03663	KEGG					
S00001	L-Homocysteine [C00155]	KEGG					
Glycine [C00037]	R00364	KEGG					
R01221	NH3 [C00014]	KEGG					
R01221 (reverse)	Glycine [C00037]	KEGG					
R00751	Glycine [C00037]	KEGG					
R00365	NH3 [C00014]	KEGG					
R00581	NH3 [C00014]	KEGG					
NH3 [C00014]	R00366 (reverse)	KEGG					
interleukin 1 receptor, type I precursor [Homo sapiens]	interleukin 1, beta proprotein [Homo sapiens]	BioGrid;BIND		Invitro;x-ray: x-ray crystallography			9062193
L-Serine [C00065]	R00220	KEGG					
R00364 (reverse)	Glycine [C00037]	KEGG					
HCLS1 associated protein X-1 isoform b [Homo sapiens]	interleukin 1, alpha proprotein [Homo sapiens]	BioGrid;BioGrid		Invivo			11554782
glycodelin precursor [Homo sapiens]	heat shock 70kDa protein 1B [Homo sapiens]	IntAct			MI:0398 two hybrid pooling approach	MI:0218 physical interaction	16169070
L-Serine [C00065]	R00581	KEGG					
NH3 [C00014]	R00996 (reverse)	KEGG					
L-Serine [C00065]	R03389	KEGG					

O-Phospho-L-homoserine [C01102]	S00001	KEGG					
R03389	Glycine [C00037]	KEGG					
R03389 (reverse)	L-Serine [C00065]	KEGG					
R01290 (reverse)	L-Serine [C00065]	KEGG					
TNF receptor-associated factor 2 [Homo sapiens]	Ran binding protein 11 [Homo sapiens]	IntAct;BioGrid		;two hybrid	MI:0398 two hybrid pooling approach;	MI:0218 physical interaction;	16189514
R01290 (reverse)	L-Homocysteine [C00155]	KEGG					
L-Threonine [C00188]	R00751	KEGG					
glycodelin precursor [Homo sapiens]	alpha-2-macroglobulin precursor [Homo sapiens]	BioGrid		Invitro			11023837
4.3.1.19 Threonine ammonia-lyase	R00220 (reverse)	KEGG					
tumor necrosis factor alpha [Homo sapiens]	TNF receptor-associated factor 2 [Homo sapiens]	Reactome indirect_complex;IntAct;BioBridge textmining			MI:0007 anti tag coimmunoprecipitation	MI:0218 physical interaction	12887920
TAT_HV1H2	tumor necrosis factor alpha [Homo sapiens]						
L-Threonine [C00188]	R03663	KEGG					
ADP-ribosylation factor 6 [Homo sapiens]	Ran binding protein 11 [Homo sapiens]	IntAct			MI:0006 anti bait coimmunoprecipitation	MI:0218 physical interaction	17353931
Glycine [C00037]	R00366	KEGG					
alpha-2-macroglobulin precursor [Homo sapiens]	interleukin 1, beta proprotein [Homo sapiens]	BioGrid		Invitro			9714181
L-Serine [C00065]	R00588	KEGG					
tumor necrosis factor receptor 1 precursor [Homo sapiens]	HCLS1 associated protein X-1 isoform b [Homo sapiens]	IntAct			MI:0676 tandem affinity purification	MI:0218 physical interaction	14743216
R00751 (reverse)	L-Threonine [C00188]	KEGG					
R00945	L-Serine [C00065]	KEGG					
NH3 [C00014]	R00365 (reverse)	KEGG					
tumor necrosis factor receptor 1 precursor [Homo sapiens]	heat shock 70kDa protein 1B [Homo sapiens]	IntAct			MI:0676 tandem affinity purification;MI:0676 tandem affinity purification;MI:0676 tandem affinity purification	MI:0218 physical interaction	14743216
R00996	NH3 [C00014]	KEGG					

tumor necrosis factor receptor 2 precursor [Homo sapiens]	HCLS1 associated protein X-1 isoform b [Homo sapiens]	IntAct			MI:0676 tandem affinity purification	MI:0218 physical interaction	14743216
TAT_HV1H2	interleukin 1, beta proprotein [Homo sapiens]						
phytanoyl-CoA hydroxylase interacting protein [Homo sapiens]	S100 calcium binding protein A13 [Homo sapiens]	IntAct			MI:0398 two hybrid pooling approach	MI:0218 physical interaction	16169070
interleukin 1 receptor, type I precursor [Homo sapiens]	TNF receptor-associated factor 6 [Homo sapiens]	IntAct			MI:0006 anti bait coimmunoprecipitation	MI:0218 physical interaction	16286467
Glycine [C00037]	R00365	KEGG					
R00220	NH3 [C00014]	KEGG					
R01466 (reverse)	O-Phospho-L-homoserine [C01102]	KEGG					
NH3 [C00014]	R00581 (reverse)	KEGG					
tumor necrosis factor receptor 1 precursor [Homo sapiens]	hydroxyacyl dehydrogenase, subunit A [Homo sapiens]	IntAct			MI:0676 tandem affinity purification	MI:0218 physical interaction	14743216
TNF receptor-associated factor 6 [Homo sapiens]	threonyl-tRNA synthetase [Homo sapiens]	IntAct			MI:0006 anti bait coimmunoprecipitation	MI:0218 physical interaction	17353931
R00588	Glycine [C00037]	KEGG					
interleukin 1, alpha proprotein [Homo sapiens]	interleukin 1, beta proprotein [Homo sapiens]	ProLink	0.663		GC Gene Cluster		
